# Petrological and geochemical (major-, trace-, and rare earth element) data of the Triassic El Tranquilo Group, Deseado Massif, Patagonia, Argentina

**DOI:** 10.1016/j.dib.2018.11.062

**Published:** 2018-11-16

**Authors:** Uwe Jenchen

**Affiliations:** Uwe Jenchen, Universidad Autónoma de Nuevo León, Facultad de Ciencias de la Tierra, Carretera a Cerro Prieto, km. 8, C.P. 67700 Linares, Nuevo León, México

## Abstract

From samples of the Middle to Late Triassic El Tranquilo Group (El Tranquilo anticline, Deseado Massif, Patagonia) petrographic (qualitative and modal) analyses and geochemical analyses (major, trace elements, and rare earth elements (REEs)) of 80 samples were carried out. The data presented here contain a broad overview of photomicrography, recalculated modal point-count data, geochemical raw data, and simple statistics of selected geochemical parameters. The data presented in this article are interpreted and discussed in the research article entitled “Petrography and geochemistry of the Triassic El Tranquilo Group, Deseado Massif, Patagonia, Argentina: Implications for provenance and tectonic setting” (Jenchen, 2018).

**Specifications table**

**Table:** 

**Subject area**	*Earth Sciences*
**More specific subject area**	*Petrology, Geochemistry*
**Type of data**	*Microscopy images, tables, figures and graphs*
**How data was acquired**	*Major and trace element concentrations were determined on these sample tablets (Major elements as oxides in weight %, trace elements in ppm) using a sequentially operating, wavelength-dispersive X-ray fluorescence spectrometer (SIEMENS SRS 303 AS) on a volatile-free base. Rare Earth Elements was conducted by atomic emission spectroscopy, using inductively coupled plasma excitation at an ICP-AES (Jobin YVON Model 38 plus;*Table 10, Table 11, Table 12, Table 13, Table 14, Table 15*). 35 samples were analyzed and pulverized and by ICP-ES (oxides, Ba, Ni, Sc), and ICP-MS (trace and rare-earth elements) at ACME Laboratories, Vancouver, Canada.*
**Data format**	*Raw (photos), Analyzed, processed and filtered*
**Experimental factors**	*Thin sections were prepared, point-counted and photographed. 34 samples were crushed, pulverized, LOI (loss on ignition, the pre-annealed material was mixed with lithium tetraborate (Li2B4O7) in the ratio 2:1, melted at 1,400 ° C in a graphite crucible and poured into platinum pouring bowls.*
**Experimental features**	*Petrological and geochemical analysis of the rocks of The El Tranquilo Anticline*
**Data source location**	*El Tranquilo Anticline, Deseado Massif, Patagonia, Argentina*
**Data accessibility**	*Data available within this article*
**Related research article**	*Jenchen, U (2018).Petrography and geochemistry of the Triassic El Tranquilo Group, Deseado Massif, Patagonia, Argentina: Implications for provenance and tectonic setting. Journal of South American Earth Sciences, 88: 530-550. –*https://doi.org/10.1016/j.jsames.2018.09.007[1]

**Value of the data**

• Determine to the lithological and geochemical characteristics of the working area.
• Tectonic activity, weathering, and provenance of the El Tranquilo Group.
• Data collection available for researches working in the Western Margin of Gondwana, and adjacent areas.
• Data collection available for sedimentologists, working with geochemical data.
• A most complete geochemical dataset for El Tranquilo Group.

## 1 Data

This article provides photomicrographies from sedimentary and igneous rocks, recalculated petrographic modal, analyses and geochemical analyses (major, trace elements, and rare earth elements (REEs)) of 80 samples. The sample location is given with the geographical coordinates of each sample and with its position in the stratigraphic column. The geochemical are presented as raw data, and simple statistic of selected geochemical parameters. Additional contains CIA, Ti/Nb-ratios, SiO_2_/K_2_O-ratios values of geochemical standards used for comparison in Fig. 10, Fig. 16 (recalculated data from [8]).

## 2 Experimental design, materials and methods

**Fig. 1:**
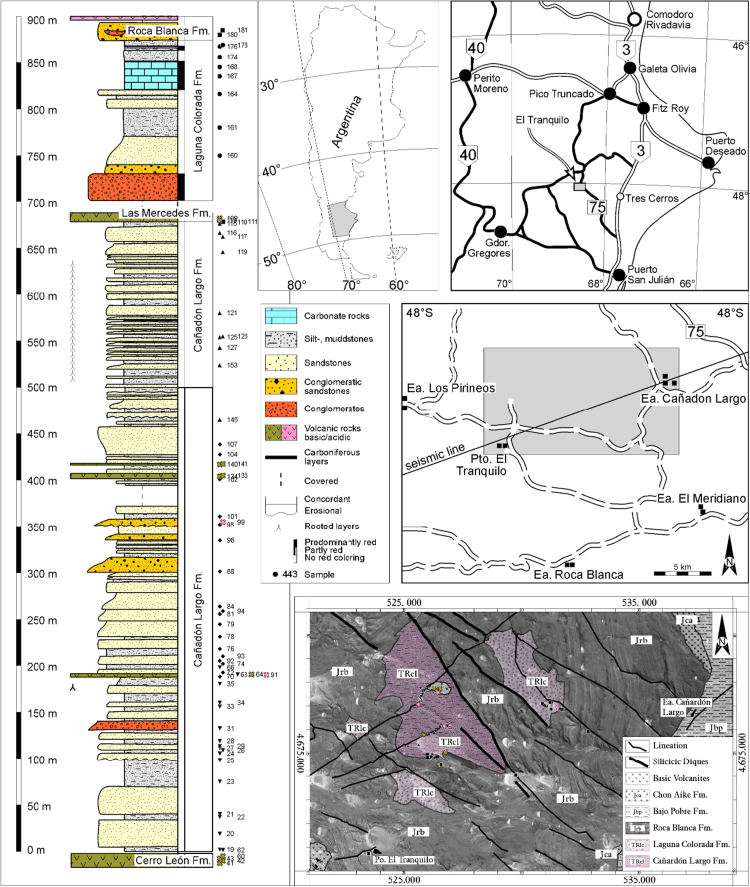
Position and simplified geological map with and sample location (right) displayed on a Google Earth image [2]; stratigraphic column and locations of the samples used for this data collection (modified after [1]; left).

Field work was carried out from −January 21 to February 4, 1991. Cartographic basis for the field work comprised Servicio Geológico Nacional topographical maps at a scale of 1:100,000; in 2016, the sample sites were located in Google Earth Pro (2016) sample sites were located in Google Earth Pro (2016) with a precision of +10 m. A detailed description of sampling and sample processing is given in [1] (Fig. 1 and Table 1).

**Table 1:** Sample list and sample locations.

**Sample**	**Lithology**	**m**	**Formation**	**UTM-E**	**UTM-N**	**Latitude (°N)**	**Longitude (°E)**
**ET-19**	T	2	Cañadón Largo	19-F-525.994	4.677.721	−48,05326 °N	−68,65118 °E
**ET-20**	Cgl	19	Cañadón Largo	19-F-525.937	4.677.700	−48,05342 °N	−68,65194 °E
**ET-21**	T	40	Cañadón Largo	19-F-525.867	4.677.643	−48,05397 °N	−68,65286 °E
**ET-22**	U	37	Cañadón Largo	19-F-525.875	4.677.653	−48,05388 °N	−68,65277 °E
**ET-23**	U	75	Cañadón Largo	19-F-525.791	4.677.484	−48,05540 °N	−68,65388 °E
**ET-24**	S	105.5	Cañadón Largo	19-F-525.729	4.677.384	−48,05000 °N	−69,20000 °E
**ET-25**	U	98	Cañadón Largo	19-F-525.742	4.677.411	−48,05606 °N	−68,65454 °E
**ET-26**	tS	109	Cañadón Largo	19-F-525.709	4.677.339	−48,65498 °N	−68,65498 °E
**ET-27**	U	111.3	Cañadón Largo	19-F-525.698	4.677.323	−48,05686 °N	−68,65512 °E
**ET-28**	sT	119	Cañadón Largo	19-F-525.696	4.677.304	−48,05703 °N	−68,65515 °E
**ET-29**	S	113.5	Cañadón Largo	19-F-525.700	4.677.290	−48,05715 °N	−68,65509 °E
**ET-29**	T	113.5	Cañadón Largo	19-F-525.700	4.677.290	−48,05715 °N	−68,65509 °E
**ET-31**	U	132,5	Cañadón Largo	19-F-525.722	4.677.260	−48,05742 °N	−68,65480 °E
**ET-32**	U	145.5	Cañadón Largo	19-F-525.724	4.677.200	−48,05796 °N	−68,65477 °E
**ET-33**	T	156	Cañadón Largo	19-F-525.700	4.677.183	−48,05812 °N	−68,65509 °E
**ET-34**	S-Carb	160	Cañadón Largo	19-F-525.688	4.677.170	−48,05817 °N	−68,65525 °E
**ET-35**	T	180.3	Cañadón Largo	19-F-525.649	4.677.136	−48,05854 °N	−68,65577 °E
**ET-38**	Tuf		Volcanics	19-F-526.430	4.677.570	−48,05460 °N	−68,64531 °E
**ET-41**	B	−12.5	Cerro León	19-F-526.543	4.677.734	−48,05312 °N	−68,64381 °E
**ET-42**	B	−10	Cerro León	19-F-526.527	4.677.696	−48,05347 °N	−68,64402 °E
**ET-43**	B	−7.5	Cerro León	19-F-526.496	4.677.647	−48,05391 °N	−68,64443 °E
**ET-44**	Br		Cañadón Largo	19-F-526.278	4.677.634	−48,05403 °N	−68,64736 °E
**ET-45**	U		Cañadón Largo	19-F-526.320	4.677.610	−48,05425 °N	−68,64679 °E
**ET-47**	GS		Cañadón Largo	19-F-526.362	4.677.585	−48,05447 °N	−68,64623 °E
**ET-53**	T		Cañadón Largo	19-F-525.811	4.677.527	−48,05502 °N	−68,65362 °E
**ET-57**	T		Cañadón Largo	19-F-525.716	4.677.239	−48,05761 °N	−68,65488 °E
**ET-59**	T		Cañadón Largo	19-F-525.708	4.677.223	−48,05775 °N	−68,65498 °E
**ET-60**	B	−5	Cerro León	19-F-526.290	4.677.757	−48,05293 °N	−68,64721 °E
**ET-62**	FS	1.5	Cañadón Largo	19-F-526.190	4.677.660	−48,05380 °N	−68,64854 °E
**ET-63**	T	190.3	Cañadón Largo	19-F-525.628	4.677.101	−48,05886 °N	−68,65605 °E
**ET-64**	V	190.5	Volcanics	19-F-525.615	4.677.083	−48,05902 °N	−68,65622 °E
**ET-66**	U	198	Cañadón Largo	19-F-525.613	4.677.078	−48,05906 °N	−68,65625 °E
**ET-67**	V		Cañadón Largo	19-F-525.613	4.677.070	−48,05914 °N	−68,65625 °E
**ET-68**	U		Cañadón Largo	19-F-526.346	4.675.969	−48,06902 °N	−68,64634 °E
**ET-70**	U	188	Cañadón Largo	19-F-526.933	4.676.222	−48,06671 °N	−68,63848 °E
**ET-71**	S	189	Cañadón Largo	19-F-526.944	4.676.210	−48,06678 °N	−68,63833 °E
**ET-72**	S	192.5	Cañadón Largo	19-F-526.954	4.676.200	−48,06691 °N	−68,63820 °E
**ET-74**	T	201.8	Cañadón Largo	19-F-526.938	4.676.167	−48,06720 °N	−68,63841 °E
**ET-76**	FS	218	Cañadón Largo	19-F-526.852	4.676.162	−48,06725 °N	−68,63956 °E
**ET-78**	Mg	231.5	Cañadón Largo	19-F-526.820	4.676.164	−48,06724 °N	−68,63999 °E
**ET-79**	MS	245	Cañadón Largo	19-F-526.739	4.676.154	−48,06733 °N	−68,64108 °E
**ET-81**	T	255.9	Cañadón Largo	19-F-526.702	4.676.144	−48,06742 °N	−68,64158 °E
**ET-84**	FS	264	Cañadón Largo	19-F-526.674	4.676.144	−48,06742 °N	−68,64195 °E
**ET-88**	S	301.5	Cañadón Largo	19-F-526.536	4.676.121	−48,06764 °N	−68,64380 °E
**ET-91**	V	190	Roca Blanca	19-F-526.963	4.676.190	−48,06700 °N	−68,63808 °E
**ET-92**	Cgl-cl	205	Cañadón Largo	19-F-526.928	4.676.160	−48,06723 °N	−68,63854 °E
**ET-93**	S-Carb	210	Cañadón Largo	19-F-526.868	4.676.160	−48,06722 °N	−68,63935 °E
**ET-94**	S	259.5	Cañadón Largo	19-F-526.653	4.676.140	−48,06746 °N	−68,64223 °E
**ET-96**	sT	335.5	Cañadón Largo	19-F-526.786	4.676.145	−48,06741 °N	−68,64045 °E
**ET-98**	FC	352	Cañadón Largo	19-F-526.720	4.676.151	−48,06736 °N	−68,64133 °E
**ET-99**	V	355	Roca Blanca	19-F-526.710	4.676.152	−48,06735 °N	−68,64147 °E
**ET-101**	tS	361.5	Cañadón Largo	19-F-526.688	4.676.142	−48,06744 °N	−68,64175 °E
**ET-102**	T	401	Cañadón Largo	19-F-526.276	4.675.844	−48,07014 °N	−68,64728 °E
**ET-104**	U	427.5	Cañadón Largo	19-F-526.058	4.675.795	−48,07059 °N	−68,65020 °E
**ET-107**	T	438.5	Cañadón Largo	19-F-525.997	4.675.778	−48,07074 °N	−68,65102 °E
**ET-109**	B	682	Cerro León	19-F-526.574	4.674.504	−48,08218 °N	−68,64319 °E
**ET-110**	T	678	Las Mercedes basalt	19-F-526.573	4.674.495	−48,08226 °N	−68,64320 °E
**ET-111**	T	678.5	Las Mercedes basalt	19-F-526.573	4.674.489	−48,08232 °N	−68,64320 °E
**ET-113**	B	680	Las Mercedes basalt	19-F-526.762	4.675.039	−48,07736 °N	−68,64070 °E
**ET-115**	sT	676.5	Cañadón Largo	19-F-526.773	4.675.048	−48,07728 °N	−68,64055 °E
**ET-116**	MS	665	Cañadón Largo	19-F-526.765	4.675.048	−48,07728 °N	−68,64066 °E
**ET-117**	MS	662.5	Cañadón Largo	19-F-526.774	4.675.061	−48,07716 °N	−68,64054 °E
**ET-118**	S-Carb	650	Cañadón Largo	19-F-526.754	4.675.050	−48,07718 °N	−68,64081 °E
**ET-119**	FS	646	Cañadón Largo	19-F-526.752	4.675.071	−48,07707 °N	−68,64084 °E
**ET-121**	U	580	Cañadón Largo	19-F-526.245	4.674.951	−48,07817 °N	−68,64764 °E
**ET-122**	S-Cgl	570	Cañadón Largo	19-F-526.209	4.674.960	−48,07801 °N	−68,64812 °E
**ET-125**	sT	554	Cañadón Largo	19-F-526.190	4.674.973	−48,07798 °N	−68,64838 °E
**ET-126**	U	555	Cañadón Largo	19-F-526.196	4.674.980	−48,07789 °N	−68,64830 °E
**ET-127**	U	542.8	Cañadón Largo	19-F-526.184	4.674.990	−48,07783 °N	−68,648458 °E
**ET-132**	FS		Roca Blanca	19-F-526.416	4.674.848	−48,07909 °N	−68,64533 °E
**ET-134**	B	404	Cañadón Largo	19-F-525.844	4.675.784	−48,07070 °N	−68,65307 °E
**ET-135**	V	405	Cañadón Largo	19-F-525.836	4.675.782	−48,07071 °N	−68,65318 °E
**ET-140**	B	417	Cañadón Largo	19-F-525.780	4.675.775	−48,07078 °N	−68,65393 °E
**ET-141**	V	417.5	Cañadón Largo	19-F-525.773	4.675.769	−48,07083 °N	−68,65402 °E
**ET-143**	S	458	Cañadón Largo	19-F-525.584	4.675.420	−48,07395 °N	−68,65654 °E
**ET-145**	U	465	Cañadón Largo	19-F-525.627	4.675.357	−48,07455 °N	−68,65596 °E
**ET-147**	S		Laguna Colorada	19-F-526.158	4.674.856	−48,07903 °N	−68,64880 °E
**ET-149**	S-Tuf		Laguna Colorada	19-F-526.022	4.678.410	−48,07942 °N	−68,65062 °E
**ET-150**	tS		Cañadón Largo	19-F-526.091	4.674.805	−48,07949 °N	−68,64970 °E
**ET-151**	Tuf		Roca Blanca	19-F-526.126	4.674.789	−48,07964 °N	−68,64922 °E
**ET-153**	GS	524	Cañadón Largo	19-F-525.807	4.675.135	−48,07654 °N	−68,65353 °E
**ET-157**	U		Laguna Colorada	19-F-525.996	4.674.944	−48,07825 °N	−68,65098 °E
**ET-160**	U	750	Laguna Colorada	19-F-530.885	4.677.103	−48,05860 °N	−68,58550 °E
**ET-161**	S	780	Laguna Colorada	19-F-530.929	4.677.052	−48,05906 °N	−68,58490 °E
**ET-163**	GS	805	Laguna Colorada	19-F-530.988	4.677.010	−48,05936 °N	−68,58411 °E
**ET-164**	MS	816	Laguna Colorada	19-F-531.053	4.676.988	−48,05963 °N	−68,58323 °E
**ET-167**	MS	835	Laguna Colorada	19-F-561.179	4.676.907	−48,06035 °N	−68,58154 °E
**ET-168**	U	845	Laguna Colorada	19-F-531.255	4.676.863	−48,06075 °N	−68,58051 °E
**ET-172**	GS	881.2	Laguna Colorada	19-F-531.213	4.677.154	−48,05813 °N	−68,58110 °E
**ET-174**	T	856	Laguna Colorada	19-F-531.276	4.677.148	−44,05818 °N	−68,58025 °E
**ET-175**	Later	885	Laguna Colorada	19-F-531.542	4.676.950	−48,05991 °N	−68,57667 °E
**ET-176**	Mg	867.5	Laguna Colorada	19-F-531.557	4.676.946	−48,05998 °N	−68,57647 °E
**ET-178**	T	869	Laguna Colorada	19-F-531.623	4.676.908	−48,06032 °N	−68,57558 °E
**ET-179**	S	881.2	Laguna Colorada	19-F-531.642	4.676.888	−48,06050 °N	−68,57532 °E
**ET-180**	MS	880	Roca Blanca	19-F-531.752	4.676.853	−48,06081 °N	−68,57384 °E
**ET-181**	FC	885	Roca Blanca	19-F-532.852	4.675.913	−48,06921 °N	−68,55901 °E
**ET-183**	sT		Cañadón Largo	19-F-529.766	4.674.053	−48,08610 °N	−68,60030 °E
**ET-185**	Tuf		Roca Blanca	19-F-523.512	4.670.728	−48,11627 °N	−68,68410 °E

**Abbreviations**: B=basalt, Br=breccia, Cgl=conglomerate, Cgl-cl=conglomerate clast, FC=fine grained conglomerate, FS=fine grained sandstone, GS=coarse grained sandstone, Later=laterite, Mg=marl, MS=medium grained sandstone, S=sandstone, S-Carb=carbonate sandstone, S-Cgl= conglomeratic sandstone, sT=sandy claystone, S-Tuf=tuffitic sandstone, T=claystone, tS=muddy sandstone, Tuf=tuff, U=Silt, V=volcanics.

**Fig. 2:**
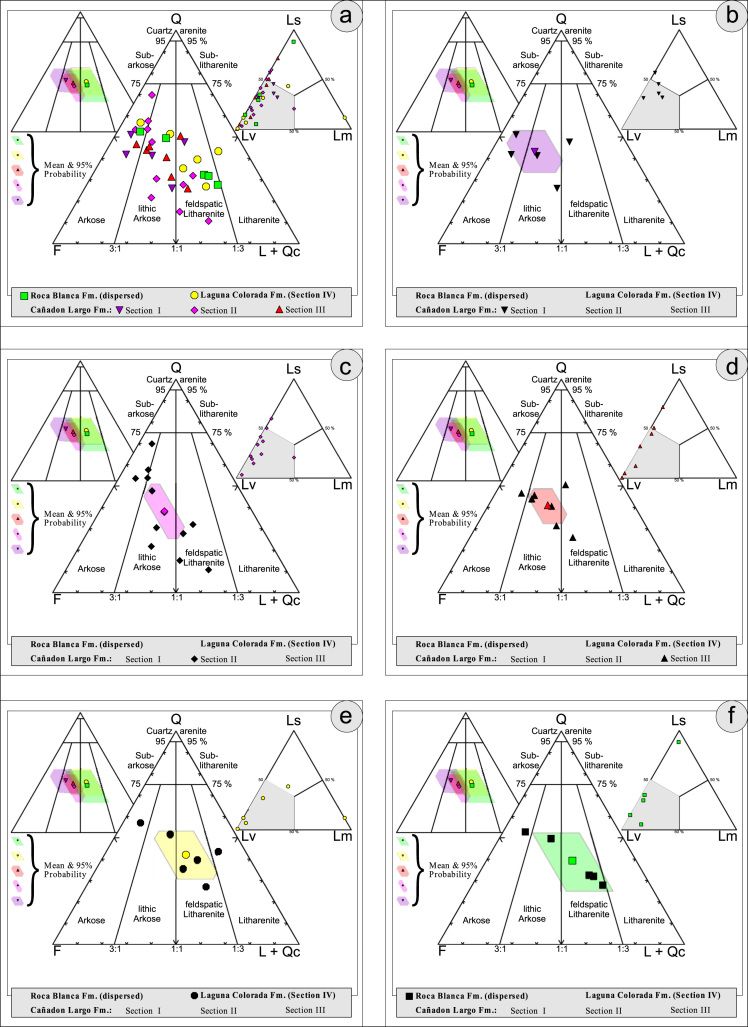
Petrographic modal analysis of El Tranquilo Group sandstones: Q–F–L after [3].

**Fig. 3:**
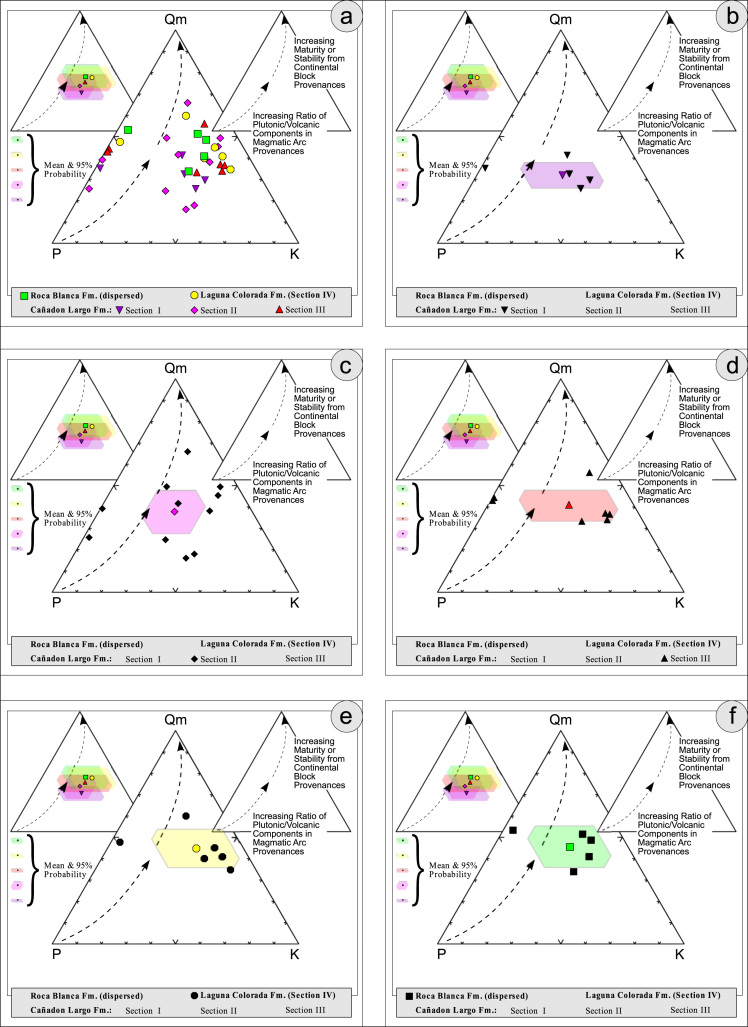
Petrographic modal analysis of El Tranquilo Group sandstones: Qm–P–K after [4].

**Fig. 4:**
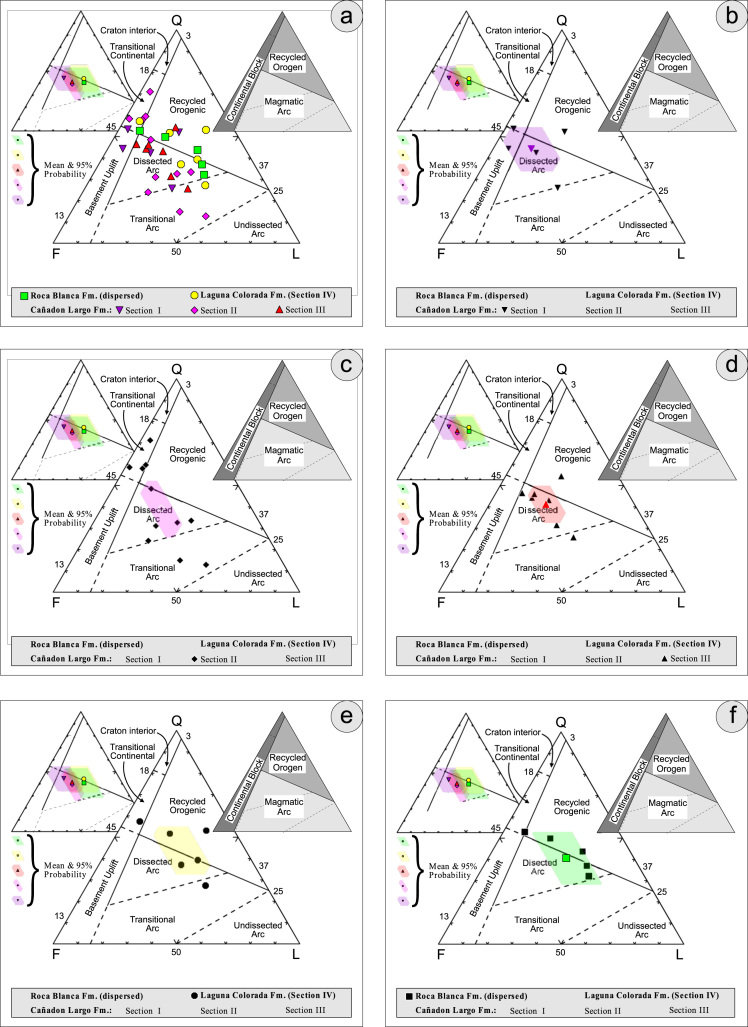
Petrographic modal analysis of El Tranquilo Group sandstones: Q–F–L after [6].

**Fig. 5:**
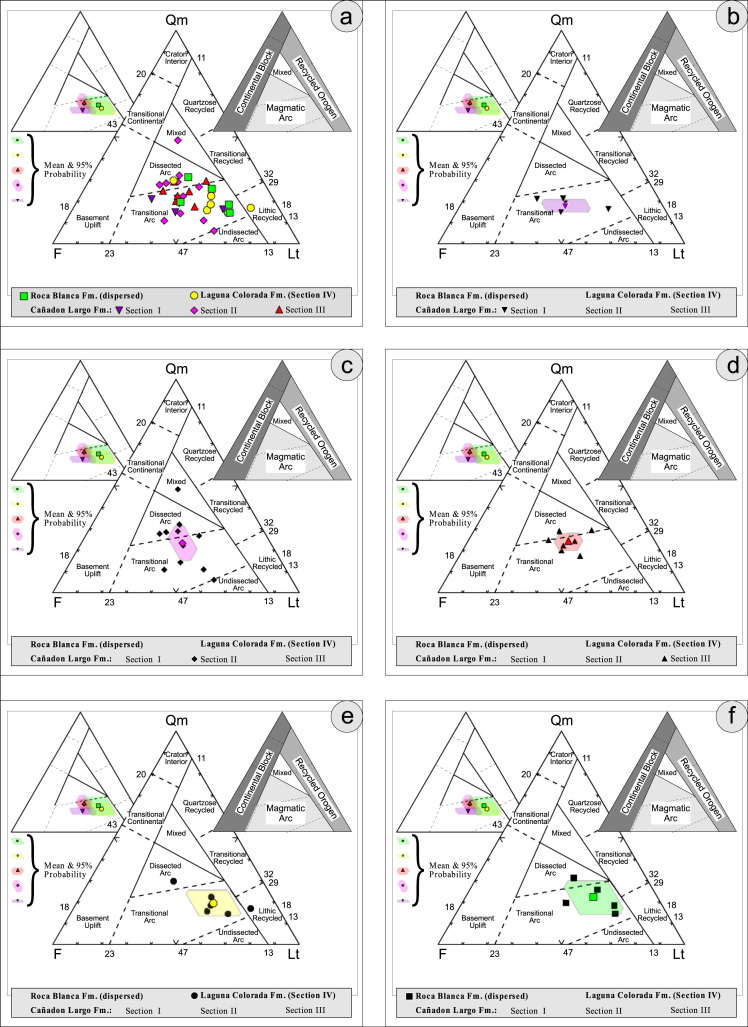
Petrographic modal analysis of El Tranquilo Group sandstones: Qm–F–Lt after [6].

**Fig. 6:**
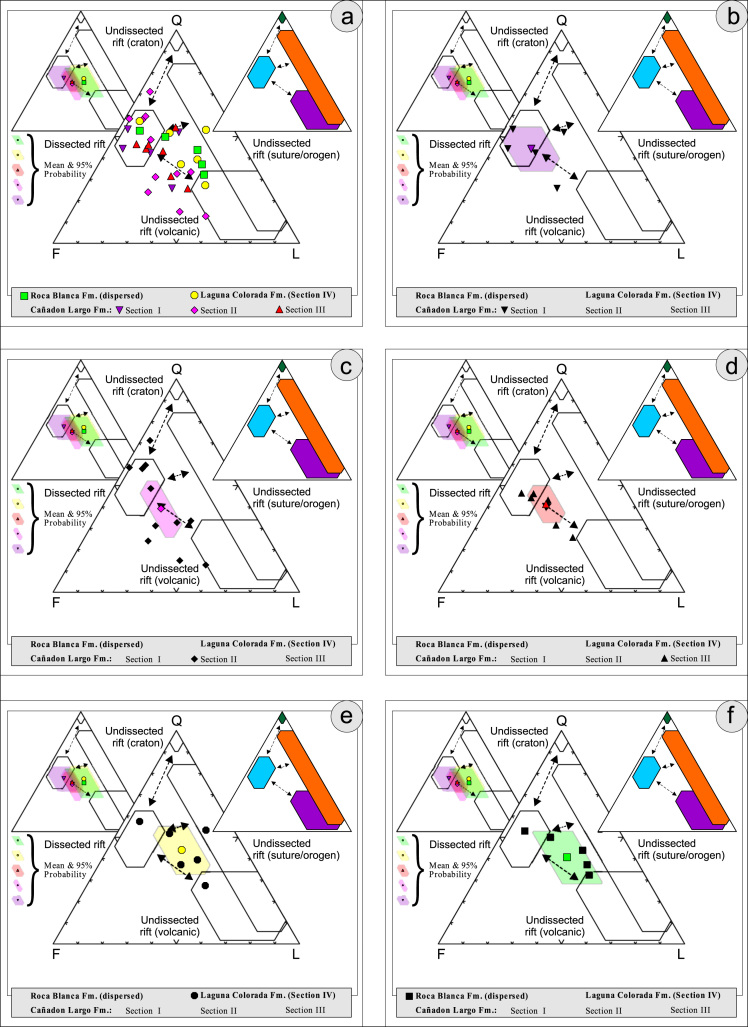
Petrographic modal analysis of El Tranquilo Group sandstones: Q–F–L diagram after [6].

Thin sections, documented in the Table 2, Table 3, Table 4, Table 5, Table 6, Table 7 are photographed using a LEICA DM2700P polarization microscope with a LEICA MC170HD Camera and a HC FL PLAN 2.5×0.07 Lens; each with parallel and crossed Nicols. Modal analyses were carried out on 37 samples, counting 300–500 points using the Gazzi–Dickinson technique to minimize the compositional dependence on grain size [17] (see Table 9, based on [3], [4], [5], [6]; see also Fig. 2, Fig. 3, Fig. 4, Fig. 5, Fig. 6). The 95 confidence intervals for Student׳s *t*-test [18] were plotted in optically distinct shades (Table 8, Table 9).

**Table 2:** Photmicrographies of thin sections from Section I (Cañadón Largo Formation).

**Table 3:** Photmicrographies of thin sections from Section II (Cañadón Largo Formation).

**Table 4:** Photmicrographies of thin sections from Section III (Cañadón Largo Formation).

**Table 5:** Photmicrographies of thin sections from Section IV (Laguna Colorada Formation).

**Table 6:** Photmicrographies of thin sections from Section V (Roca Blanca Formation).

**Table 7:** Photmicrographies of thin sections of acidic volcanic rocks.

**Table 8:** Photmicrographies of thin sections of basic igneous rocks.

**Table 9:** Recalculated modal point-count data for analyzed sandstones.

**Sample**	[3]			[3]			[4]			[5], [6]			[5]			**P/F**	**Lv/L**
	**Q**	**F**	**L+Qc**	**Ls**	**Lv**	**Lm**	**Qm**	**P**	**K**	**Q**	**F**	**L**	**Qm**	**F**	**Lt**		
**Lower Cañadón Largo Formation (Section I)**																	
ET-44	26	39	35	32	49	20	35	63	1	26	39	35	21	39	40	0.97	0.48
ET-44	26	39	35	32	49	20	35	63	1	26	39	35	21	39	40	0.97	0.48
ET-20	48	23	30	31	66	3	41	27	32	50	24	26	16	23	61	0.45	0.65
ET-24	51	43	6	57	43	0	26	29	45	52	44	4	15	43	42	0.39	0.42
ET-29	42	49	9	45	45	9	30	23	47	43	51	6	21	49	30	0.33	0.45
ET-34	51	43	6	57	43	0	26	29	45	52	44	4	15	43	42	0.39	0.42
ET-63	41	39	20	36	50	14	33	30	37	42	40	19	19	39	42	0.44	0.49
**Mean (AM)**	**43.2**	**39.3**	**17.7**	**43.0**	**49.3**	**7.7**	**31.8**	**33.5**	**34.5**	**44.2**	**40.3**	**15.7**	**17.8**	**39.3**	**42.8**	**0.50**	**0.49**
**Confidence (**−**95%)**	**33.2**	**30.1**	**4.3**	**30.5**	**40.2**	−**0.9**	**25.8**	**18.1**	**16.3**	**33.7**	**30.8**	**1.9**	**14.8**	**30.1**	**32.3**	**0.25**	**0.39**
**Confidence (+95%)**	**53.1**	**48.6**	**31.0**	**55.5**	**58.4**	**16.2**	**37.9**	**48.9**	**52.7**	**54.6**	**49.8**	**29.4**	**20.8**	**48.6**	**53.4**	**0.74**	**0.58**

**Middle Cañadón Largo Formation (Section II)**																	
ET-68	15	41	44	37	59	4	26	73	2	15	41	44	14	41	45	0.97	0.58
ET-71	30	43	27	50	49	1	39	60	0	31	43	26	28	43	30	0.99	0.48
ET-70	48	36	16	41	59	0	38	17	45	48	36	16	22	36	42	0.27	0.58
ET-72	22	49	29	22	68	10	18	34	48	22	51	27	11	49	40	0.4	0.68
ET-79	53	40	7	33	67	0	42	28	30	56	42	2	28	40	32	0.48	0.65
ET-88	70	25	5	20	40	40	66	12	22	71	25	4	48	25	27	0.36	0.39
ET-92	11	32	58	18	80	1	16	38	46	11	33	56	6	32	62	0.45	0.8
ET-93	28	33	39	17	79	4	25	42	33	29	35	35	11	33	56	0.55	0.78
ET-94	57	33	10	14	79	7	50	29	21	59	33	8	32	33	35	0.57	0.78
ET-98	32	27	41	3	94	3	49	7	43	32	28	40	27	27	46	0.14	0.94
ET-104	54	35	12	60	40	0	45	10	44	56	36	8	29	35	36	0.18	0.39
**Mean (AM)**	**38.2**	**35.8**	**26.2**	**28.6**	**64.9**	**6.4**	**37.6**	**31.8**	**30.4**	**39.1**	**36.6**	**24.2**	**23.3**	**35.8**	**41.0**	**0.49**	**0.64**
**Confidence (**−**95%)**	**25.3**	**31.1**	**14.3**	**17.2**	**53.2**	−**1.4**	**27.4**	**17.8**	**18.7**	**25.7**	**31.7**	**12.0**	**15.1**	**31.1**	**33.7**	**0.30**	**0.52**
**Confidence (+95%)**	**51.1**	**40.5**	**38.0**	**40.1**	**76.6**	**14.2**	**47.8**	**45.9**	**42.0**	**52.4**	**41.5**	**36.3**	**31.4**	**40.5**	**48.3**	**0.68**	**0.76**

**Upper Cañadón Largo formation (Section III)**																	
ET-118	31	36	32	50	47	3	44	55	0	31	36	32	29	36	35	0.99	0.46
ET-122	26	32	42	43	53	4	43	56	1	26	33	41	25	32	43	0.98	0.53
ET-143	50	23	26	0	100	0	56	10	33	53	24	23	29	23	47	0.23	0.99
ET-116	40	34	26	32	68	0	34	14	52	42	35	24	17	34	49	0.21	0.68
ET-117	44	40	16	71	29	0	33	25	42	44	40	16	20	40	40	0.37	0.28
ET-121	46	38	16	4	96	0	37	13	49	46	38	16	22	38	39	0.21	0.95
ET-153	47	43	11	6	87	6	37	12	51	47	43	10	25	43	32	0.18	0.87
**Mean (AM)**	**40.6**	**35.1**	**24.1**	**29.4**	**68.6**	**1.9**	**40.6**	**26.4**	**32.6**	**41.3**	**35.6**	**23.1**	**23.9**	**35.1**	**40.7**	**0.45**	**0.68**
**Confidence (**−**95%)**	**32.3**	**29.1**	**14.2**	**4.4**	**43.7**	−**0.4**	**33.2**	**7.5**	**11.4**	**32.5**	**29.9**	**13.3**	**19.7**	**29.1**	**35.0**	**0.11**	**0.43**
**Confidence (+95%)**	**48.8**	**41.1**	**34.1**	**54.5**	**93.5**	**4.1**	**47.9**	**45.3**	**53.7**	**50.1**	**41.2**	**32.9**	**28.0**	**41.1**	**46.4**	**0.79**	**0.93**

**Laguna Colorada Fm.**																	
ET-163	43	11	46	0	95	5	60	16	24	49	13	38	17	11	72	0.39	0.94
ET-172	51	27	22	11	89	0	41	11	48	52	27	21	18	27	55	0.18	0.88
ET-179	39	22	39	0	0	100	40	18	42	41	23	36	14	22	64	0.3	0
ET-160	35	30	35	31	61	7	35	11	55	36	30	34	16	30	55	0.16	0.61
ET-167	27	25	49	38	37	26	48	49	3	28	26	46	22	25	53	0.93	0.36
ET-174	57	36	7	0	100	0	45	12	43	57	36	6	30	36	34	0.21	0.99
**Mean (AM)**	**42.0**	**25.2**	**33.0**	**13.3**	**63.7**	**23.0**	**44.8**	**19.5**	**35.8**	**43.8**	**25.8**	**30.2**	**19.5**	**25.2**	**55.5**	**0.36**	**0.63**
**Confidence (**−**95%)**	**30.6**	**16.3**	**16.3**	−**4.6**	**22.4**	−**17.8**	**35.7**	**4.0**	**15.8**	**32.5**	**17.8**	**15.1**	**13.4**	**16.3**	**42.1**	**0.06**	**0.22**
**Confidence (+95%)**	**53.4**	**34.0**	**49.7**	**31.3**	**104.9**	**63.8**	**53.9**	**35.0**	**55.9**	**55.2**	**33.9**	**45.2**	**25.6**	**34.0**	**68.9**	**0.67**	**1.04**

**Rincón Blanco Fm.**																	
ET-149	32	21	47	28	69	3	41	18	41	35	23	42	15	21	65	0.3	0.68
ET-132	49	29	21	14	86	0	51	16	33	50	30	21	31	29	39	0.31	0.85
ET-147	28	19	53	27	69	4	49	14	38	33	23	43	18	19	62	0.26	0.68
ET-180	52	38	9	5	81	14	34	28	38	53	38	9	20	38	42	0.42	0.8
ET-185	32	23	45	88	6	6	53	43	4	32	23	45	26	23	52	0.91	0.06
**Mean (AM)**	**38.6**	**26.0**	**35.0**	**32.4**	**62.2**	**5.4**	**45.6**	**23.8**	**30.8**	**40.6**	**27.4**	**32.0**	**22.0**	**26.0**	**52.0**	**0.44**	**0.61**
**Confidence (**−**95%)**	**24.9**	**16.5**	**11.4**	−**8.0**	**22.1**	−**1.1**	**35.8**	**8.9**	**11.9**	**28.1**	**19.1**	**12.0**	**14.0**	**16.5**	**37.6**	**0.11**	**0.22**
**Confidence (+95%)**	**52.3**	**35.5**	**58.6**	**72.8**	**102.3**	**11.9**	**55.4**	**38.7**	**49.7**	**53.1**	**35.7**	**52.0**	**30.0**	**35.5**	**66.4**	**0.77**	**1.01**

Sixty samples of El Tranquilo Group sedimentary rocks in four stratigraphic sections (Sections I: 24 samples, Section II: 18 samples, Section III: 10 samples, and section IV), eight samples, underwent geochemical analysis, along with 17 samples of co-occurring volcanic rocks and three samples of the overlying Roca Blanca Formation. A detailed description of geochemical processing and analytic methods is given in [1]. The raw and processed data are listed in the Table 10, Table 11, Table 12, Table 13, Table 14, Table 15. The distributions of the elements in the random samples were described using the arithmetic mean and confidence limits (95% and 99%, respectively) supplied by Student׳s *t*-test [18] (Table 16, Table 17) (Table 18).

**Table 10:** Geochemical parameters of samples from Section I (CaO* = recalculated CaO free of CaO in Carbonates).

**Sample**	**ET-19**	ET-20*	**ET-21**	**ET-22**	**ET-23**	ET-24*	**ET-25**	**ET-26**	**ET-27**	**ET-28**	ET-29*	ET-29*	**ET-31**	**ET-33**	ET-34*	**ET-35**	**ET-45**	**ET-47**	**ET-53**	**ET-57**	**ET-59**	**ET-62**	ET-63*	**ET-66**
**SiO2**	70.4	65.05	74.45	72.23	71.37	72.86	70.14	74.97	72.92	65.58	71.05	71.58	75.51	72.27	71.09	76.21	78.05	78.23	76.7	75.75	77.94	74.91	74.06	75.31
**TiO2**	0.59	0.39	0.42	0.45	0.51	0.36	0.6	0.37	0.52	0.79	0.55	0.45	0.35	0.3	0.39	0.42	0.49	0.26	0.49	0.42	0.25	0.34	0.25	0.5
**Al2O3**	13.9	12.09	12.45	14.12	13.78	13.25	13.99	12.55	13.2	16.56	14.63	13.49	12.16	13.67	12.53	11.9	11.23	11.3	11.16	12.04	10.3	13.37	11.3	11.4
**Fe2O3**	5.38	2.22	2.72	3.01	3.89	1.54	2.65	2.09	3.3	5.31	2.17	3.31	2.67	4.83	2.57	1.7	1.11	1.76	2.95	2.64	2.3	1.53	1.72	2.18
**MnO**	n.d.6	0.16	n.d.3	n.d.3	n.d.2	n.d.5	n.d.7	n.d.3	n.d.2	n.d.6	n.d.3	n.d.4	n.d.5	n.d.5	n.d.6	n.d.4	n.d.2	n.d.3	n.d.4	n.d.4	n.d.2	n.d.3	n.d.4	n.d.5
**MgO**	2.24	0.65	0.62	0.84	0.67	0.44	0.98	0.57	0.37	1.65	0.61	0.82	0.73	0.8	0.59	0.54	0.46	0.64	0.91	1.03	0.73	0.6	0.5	0.71
**CaO**	0.43	6.24	0.36	0.47	0.3	1.58	1.49	0.17	0.22	0.44	0.56	0.44	0.24	0.54	2.63	1	0.19	0.3	0.4	0.26	0.3	0.24	3.66	0.31
CaO*	n.d.7	4.97	n.d.	n.d.	n.d.	0.99	0.35	n.d.	n.d.	n.d.6	n.d.5	n.d.	n.d.	0.15	1.69	0.61	n.d.	n.d.	n.d.	n.d.	n.d.4	n.d.	2.99	n.d.
**Na2O**	1.1	2.79	1.17	2.48	1.55	3.42	1.82	0.73	2.57	1.9	3.3	2.42	1.73	1.08	3.89	2.35	0.4	1.58	2.05	1.92	0.98	3.01	2.76	1.84
**K2O**	2.48	3.4	5.37	4.65	5.38	3.3	4.7	6.39	5.01	4.34	3.95	3.65	4.63	2.25	2.78	2.06	6	4.4	3.26	4.13	4.07	4.5	1.91	5.92
**P2O5**	n.d.6	n.d.9	n.d.7	0.12	0.11	n.d.4	0.13	n.d.3	n.d.7	0.15	0.1	n.d.8	n.d.5	n.d.4	n.d.8	n.d.6	n.d.8	n.d.5	0.13	n.d.7	n.d.4	n.d.7	n.d.5	0.13
**LOI**	3.44	6.7	2.38	1.62	2.43	3	3.5	2.09	1.79	3.26	2.7	3.5	1.89	4.29	3.2	3.8	1.98	1.47	1.92	1.7	3.12	1.39	3.6	1.61
**CO2**	0.46	4.47	n.d.	n.d.	n.d.	1.08	0.53	n.d.	n.d.	0.27	n.d.9	n.d.	n.d.	0.35	1.74	0.84	n.d.	n.d.	n.d.	n.d.	0.13	n.d.	2.79	n.d.
CaCO3*	0.12	8.87	n.d.	n.d.	n.d.	1.77	0.62	n.d.	n.d.	0.11	n.d.9	n.d.	n.d.	0.27	3.02	1.09	n.d.	n.d.	n.d.	n.d.	n.d.7	n.d.	5.34	n.d.
**Cr**	19	27	14	23	17	0	31	13	14	49	20	20	10	9	20	7	18	10	13	29	8	11	13	16
**Ni**	0	8	3	11	0	4	13	0	0	14	5	10	1	0	13	0	1	14	0	11	0	0	10	0
**Co**	7	4	10	10	10	2	16	9	14	12	3	5	8	7	5	3	11	9	9	9	7	19	4	14
**Sc**	15	6	11	6	8	4	11	6	12	17	5	8	9	9	6	10	4	4	6	10	7	5	4	7
**V**	58	48	46	49	51	23	47	46	81	89	30	56	37	31	79	31	45	32	40	58	26	38	26	45
**Pb**	42	12	45	30	48	13	68	56	83	52	15	28	52	39	15	37	46	14	36	38	31	49	13	55
**Zn**	77	31	73	70	61	47	71	23	45	111	82	97	61	24	61	27	22	34	57	42	15	29	24	58
**Rb**	144	111	209	185	213	119	180	257	199	198	144	145	186	145	89	118	290	187	143	173	194	136	77	214
**Ba**	420	1428	896	1056	1177	671	1265	1743	1275	997	1049	962	1264	746	10053	1517	878	925	1230	1115	1065	1083	370	2284
**Sr**	139	302	127	186	121	179	200	115	181	148	203	155	134	210	248	374	217	364	171	180	129	180	283	146
**Ga**	22	13	17	16	19	15	18	16	17	22	15	15	16	21	10	14	15	9	16	16	15	13	10	15
**Ta**	1.06	0.5	1.41	2.37	1.14	0.8	2.05	1.33	1.4	0.85	1	0.8	1.51	0.94	0.5	0.97	1.85	1.89	1.79	1.31	1.16	1.7	0.6	1.63
**Nb**	13.2	7.9	11.3	15.8	14.1	13.5	17.7	10.3	15.1	17.9	16.9	12.2	9.7	14.6	8.9	11.8	11.2	12.8	13.4	10.3	6.9	10.1	6.3	12.9
**Hf**	8.47	3.6	9.55	4.78	10.1	4.9	11.35	9.18	14.05	10.1	15.3	4.9	9	9.58	4.8	12.65	9.68	4.11	10.5	9.27	8.87	10.9	2.9	11.55
**Zr**	92	138	108	92	145	184	191	116	246	170	587	168	109	131	193	192	97	139	135	106	90	156	101	174
**Y**	19	14	21	29	33	21	40	21	23	35	30	21	21	25	15	25	33	17	28	18	17	20	16	28
**Th**	10.25	10.5	16.6	15.3	20.45	15.6	24.75	20.25	22.6	20.35	33.4	18.3	14.55	14.55	13.7	8.99	20.25	13.55	12.55	13.7	12.95	15.1	8	23
**U**	2.08	1.8	2.49	2.94	3.82	2.3	2.61	2.61	2.94	3.78	4.3	4.2	2.04	2.41	2.5	3.05	3.45	2.76	2.38	3.42	2.83	3.15	1.9	4.17
**La**	n.d.	37.4	n.d.	46.43	n.d.	60.8	n.d.	n.d.	n.d.	n.d.	87.3	35.1	n.d.	n.d.	43.3	n.d.	n.d.	n.d.	n.d.	n.d.	n.d.	n.d.	32.6	n.d.
**Ce**	n.d.	76.2	n.d.	92.72	n.d.	121.3	n.d.	n.d.	n.d.	n.d.	175.9	64.9	n.d.	n.d.	82.7	n.d.	n.d.	n.d.	n.d.	n.d.	n.d.	n.d.	58.9	n.d.
**Pr**	n.d.	8.29	n.d.	10.15	n.d.	12.74	n.d.	n.d.	n.d.	n.d.	18.78	7.38	n.d.	n.d.	8.62	n.d.	n.d.	n.d.	n.d.	n.d.	n.d.	n.d.	6.59	n.d.
**Nd**	n.d.	29.9	n.d.	39.31	n.d.	44.1	n.d.	n.d.	n.d.	n.d.	71.9	24.7	n.d.	n.d.	31.1	n.d.	n.d.	n.d.	n.d.	n.d.	n.d.	n.d.	23.3	n.d.
**Sm**	n.d.	4.76	n.d.	9.47	n.d.	7.7	n.d.	n.d.	n.d.	n.d.	11.28	4.81	n.d.	n.d.	4.96	n.d.	n.d.	n.d.	n.d.	n.d.	n.d.	n.d.	3.76	n.d.
**Eu**	n.d.	0.81	n.d.	2.44	n.d.	1.3	n.d.	n.d.	n.d.	n.d.	1.81	1.04	n.d.	n.d.	0.98	n.d.	n.d.	n.d.	n.d.	n.d.	n.d.	n.d.	1	n.d.
**Gd**	n.d.	3.98	n.d.	10.64	n.d.	5.66	n.d.	n.d.	n.d.	n.d.	9.05	4.28	n.d.	n.d.	3.83	n.d.	n.d.	n.d.	n.d.	n.d.	n.d.	n.d.	3.42	n.d.
**Tb**	n.d.	0.54	n.d.	1.41	n.d.	0.76	n.d.	n.d.	n.d.	n.d.	1.18	0.69	n.d.	n.d.	0.53	n.d.	n.d.	n.d.	n.d.	n.d.	n.d.	n.d.	0.52	n.d.
**Dy**	n.d.	2.52	n.d.	8.35	n.d.	4.39	n.d.	n.d.	n.d.	n.d.	6.3	4.27	n.d.	n.d.	2.87	n.d.	n.d.	n.d.	n.d.	n.d.	n.d.	n.d.	2.86	n.d.
**Ho**	n.d.	0.45	n.d.	n.d.	n.d.	0.74	n.d.	n.d.	n.d.	n.d.	1.01	0.85	n.d.	n.d.	0.55	n.d.	n.d.	n.d.	n.d.	n.d.	n.d.	n.d.	0.54	n.d.
**Er**	n.d.	1.36	n.d.	4.65	n.d.	1.79	n.d.	n.d.	n.d.	n.d.	2.97	2.4	n.d.	n.d.	1.67	n.d.	n.d.	n.d.	n.d.	n.d.	n.d.	n.d.	1.66	n.d.
**Tm**	n.d.	0.24	n.d.	n.d.	n.d.	0.28	n.d.	n.d.	n.d.	n.d.	0.44	0.38	n.d.	n.d.	0.26	n.d.	n.d.	n.d.	n.d.	n.d.	n.d.	n.d.	0.19	n.d.
**Yb**	n.d.	1.43	n.d.	4.85	n.d.	1.6	n.d.	n.d.	n.d.	n.d.	2.73	2.49	n.d.	n.d.	1.75	n.d.	n.d.	n.d.	n.d.	n.d.	n.d.	n.d.	1.59	n.d.
**Lu**	n.d.	0.2	n.d.	0.84	n.d.	0.29	n.d.	n.d.	n.d.	n.d.	0.44	0.38	n.d.	n.d.	0.27	n.d.	n.d.	n.d.	n.d.	n.d.	n.d.	n.d.	0.21	n.d.
**Chem.Lit**	Psam.	CaO++	Psam.	Rest++	Psam.	Psam.	Psam.	Psam.	Psam.	Pelite	Rest++	Psam.	Psam.	Psam.	Psam.	Psam.	Psam.	Psam.	Psam.	Psam.	Psam.	Psam.	Psam.	Psam.
**Zr/Ti**	155.93	355.38	257.14	204.44	284.31	513.05	318.33	313.51	473.07	215.18	1068.54	374.44	311.42	436.66	496.66	457.14	197.95	534.61	275.51	252.38	360	458.82	404	348
**Nb/Y**	0.69	0.56	0.53	0.54	0.42	0.63	0.44	0.49	0.65	0.51	0.54	0.56	0.46	0.58	0.57	0.47	0.33	0.75	0.47	0.57	0.4	0.5	0.39	0.46
**Th/Sc**	0.68	1.75	1.5	2.55	2.55	3.9	2.25	3.37	1.88	1.19	6.68	2.28	1.61	1.61	2.28	0.89	5.06	3.38	2.09	1.37	1.85	3.02	2	3.28
**Ti/Nb**	268	296	223	171	217	160	203	215	206	265	195	221	216	123	263	213	262	122	219	244	217	202	238	232
**CIA**	73.5	53.8	60.2	59.2	61.3	56.6	58.6	60.	57.1	66.9	58.4	61.2	59.8	73.9	53.4	64.1	60.5	59.2	60.3	60.2	61.7	56.9	59.4	54
**PIA**	82.8	55.7	73.2	66	73.6	59.4	65	79.7	63.4	77.3	62.8	67.4	69.3	82.4	54.5	68.5	84.9	68.3	66.6	68.5	74.7	61.8	62	60.1
**CIW**	85.6	64.3	83.6	75.1	82.8	66.7	74.5	89.7	74.7	82.6	70.4	74.5	79.3	85.1	61.2	72.8	93	78.8	74.5	77.5	83.8	71.8	66.7	77.5
Eu/Eu*	n.d.	0.57	n.d.	0.74	n.d.	0.6	n.d.	n.d.	n.d.	n.d.	0.55	0.7	n.d.	n.d.	0.69	n.d.	n.d.	n.d.	n.d.	n.d.	n.d.	n.d.	0.85	n.d.
**REE**	n.d.	17.67	n.d.	6.47	n.d.	25.68	n.d.	n.d.	n.d.	n.d.	21.61	9.53	n.d.	n.d.	16.72	n.d.	n.d.	n.d.	n.d.	n.d.	n.d.	n.d.	13.85	n.d.
**LREE**	n.d.	4.95	n.d.	3.09	n.d.	4.97	n.d.	n.d.	n.d.	n.d.	4.87	4.59	n.d.	n.d.	5.49	n.d.	n.d.	n.d.	n.d.	n.d.	n.d.	n.d.	5.46	n.d.
**HREE**	n.d.	2.26	n.d.	1.78	n.d.	2.87	n.d.	n.d.	n.d.	n.d.	2.69	1.39	n.d.	n.d.	1.77	n.d.	n.d.	n.d.	n.d.	n.d.	n.d.	n.d.	1.74	n.d.
**S-REE**	n.d.	168.08	n.d.	231.26	n.d.	263.45	n.d.	n.d.	n.d.	n.d.	391.09	153.67	n.d.	n.d.	183.39	n.d.	n.d.	n.d.	n.d.	n.d.	n.d.	n.d.	137.14	n.d.

Note: Oxides and LOI in %, other elements in ppm. X is mean value for each group of sandstones; SD is standard derivation for that mean. Abbreviations: n.d.: not detected; CaCO* = maximum CaO in Carbonates recalculated from CO_2_; Chem.Lit: Chemical lithology [7] (s. Fig. 7); CaO++: CaO enriched samples; Psam.: Psammite classified samples; Rest++: enriched in SiO_2_ and Al_2_O_3_; Rest--: impoverished in SiO_2_ and Al_2_O_3_;. Eu/Eu* = Eu_N_/(Sm_N_xGd_N_)^0.5^ Samples are not LOI-free recalculated. Samples marked with (*) analyzed by ACME Laboratories, Canada.

**Table 11:** Geochemical parameters of samples from Section II (CaO* = recalculated CaO free of CaO in Carbonates).

Sample	ET-70*	ET-72*	ET-74*	ET-76	**ET-78**	ET-79*	**ET-81**	**ET-84**	ET-88*	ET-92*	ET-93*	ET-94*	**ET-96**	ET-98*	**ET-101**	**ET-102**	**ET-104**	**ET-107**
**SiO**_**2**_	73.18	48.3	54.56	71	74.78	60.9	75.34	70.44	52.33	64.07	47.97	68.94	79.38	71.61	74.9	68.04	64.56	67.99
**TiO**_**2**_	0.33	0.34	0.41	0.55	0.53	1.01	0.47	0.58	0.31	0.6	0.46	0.51	0.37	0.54	0.53	0.41	0.62	0.33
**Al**_**2**_**O**_**3**_	13.27	10.72	13.18	14.06	13.15	16.01	11.67	13.8	12.41	15	11.68	14.7	11.3	13.33	12.44	15.15	13	8.91
**Fe**_**2**_**O**_**3**_	2.51	2.29	3.05	4.48	2.34	8.04	3.53	2.89	2.11	3.96	3.37	4.19	1.86	4.03	4.14	3.48	7.98	12.94
**MnO**	0.04	0.61	0.02	0.03	0.02	0.08	0.04	0.05	0.39	0.11	0.45	0.06	0.01	0.06	0.03	0.03	0.08	0.09
**MgO**	0.75	0.52	0.95	1.13	0.91	2.22	1.06	1.41	0.67	1.26	0.82	0.93	0.75	1.26	1.52	1.63	2.27	4.51
**CaO**	0.3	16.31	0.89	0.51	0.32	1	0.28	1.95	12.8	3.03	14.98	0.7	0.27	0.69	0.18	0.12	2.38	0.33
CaO*	n.d.	15.86	n.d.	0.08	n.d.	0.11	n.d.	0.56	12.69	1.65	13.9	0.08	n.d.	0.06	n.d.	0.05	0.7	0.11
**Na**_**2**_**O**	1.45	1.82	0.69	1.58	1.7	3.5	1.11	1.89	1.19	3.11	2.04	3.28	1.12	2.26	1.19	0.8	2.24	0.21
**K**_**2**_**O**	5.35	3.64	3	3.37	3.61	2.05	3.62	3.07	5.15	2.98	3.8	3.72	2.34	1.61	2.15	5.7	1.32	0.18
**P**_**2**_**O**_**5**_	0.02	0.07	0.02	0.12	0.16	0.18	0.14	0.18	0.09	0.13	0.1	0.09	0.1	0.15	0.06	0.06	0.11	0.11
**LOI**	2.6	15.1	23.1	3.24	2.52	4.7	2.72	3.84	12.3	5.5	14	2.7	2.54	4.2	2.93	4.8	5.2	4.63
**CO2**	n.d.	13	n.d.	0.25	n.d.	0.35	n.d.	0.88	10.86	2.05	11.74	0.19	n.d.	0.16	n.d.	1.89	1.28	1.71
**CaCO**_3_*	n.d.	28.31	n.d.	0.14	n.d.	0.2	n.d.	1	22.65	2.94	24.81	0.14	n.d.	0.11	n.d.	0.09	1.25	0.2
**Cr**	13	20	27	29	26	54	19	22	0	34	27	34	11	34	16	9	47	32
**Ni**	11	8	3	17	14	13	0	0	5	11	9	13	3	15	1	5	22	3
**Co**	10	8	2	12	10	18	9	11	5	13	12	9	5	10	6	12	17	12
**Sc**	7	6	11	13	15	17	8	11	5	13	8	9	14	7	11	10	10	10
**V**	40	57	78	72	72	197	48	55	30	108	82	147	62	86	50	39	82	73
**Pb**	18	8	22	54	36	6	53	40	10	14	8	14	32	17	33	52	13	15
**Zn**	56	35	38	86	46	87	45	47	34	52	46	88	18	67	54	97	74	71
**Rb**	229	126	139	159	159	79	163	136	181	119	126	128	129	76	103	153	51	9
**Ba**	1497	1765	510	904	1434	777	796	590	1110	898	1822	823	591	1079	393	512	806	134
**Sr**	135	348	139	136	134	225	86	352	206	301	372	187	92	325	88	79	400	65
**Ga**	17	12	13	21	16	21	22	21	13	18	13	17	18	15	19	20	15	18
**Ta**	0.7	0.6	0.5	1.19	1.84	0.9	1.94	0.78	0.6	0.6	0.4	1	1.36	0.7	0.89	0.87	0.5	0.82
**Nb**	9.1	6	7	13.1	15.6	12.8	15	16.7	7.4	8.5	6.8	9.7	9.2	9.9	12	10.2	8.2	8.1
**Hf**	2.6	3.3	4.3	9.37	4.55	9.4	9.81	13.65	3.5	5	3.5	5.9	9.49	4.6	11.75	8.37	4.6	6.8
**Zr**	74	112	157	138	138	374	117	245	126	190	121	223	98	179	170	117	167	77
**Y**	12	17	29	24	20	28	25	31	12	16	20	18	20	22	22	26	18	14
**Th**	13.3	8.9	10.9	15.5	15.65	16.9	17	19.35	9.2	13.4	10.4	17.3	10.45	13.6	12.05	17.95	8.2	3.79
**U**	2.6	1.3	3.2	3.2	2.39	2.3	3.95	4.41	1.8	2.3	1.8	3	2.3	3.6	2.94	3.62	1.9	2.13
**La**	20.5	27.5	40.5	n.d.	n.d.	59.7	n.d.	n.d.	27	40.6	39	53.7	n.d.	65	n.d.	n.d.	32.7	n.d.
**Ce**	40.8	50.9	84.7	n.d.	n.d.	121.2	n.d.	n.d.	52.4	77.9	72.6	97.3	n.d.	128.9	n.d.	n.d.	65.4	n.d.
**Pr**	4.45	5.87	9.37	n.d.	n.d.	13.22	n.d.	n.d.	5.6	8.33	7.74	10.12	n.d.	13.24	n.d.	n.d.	7.13	n.d.
**Nd**	15.7	22.1	35.2	n.d.	n.d.	48.1	n.d.	n.d.	19.5	28.9	29.7	37.2	n.d.	47	n.d.	n.d.	27	n.d.
**Sm**	2.58	4.1	6.82	n.d.	n.d.	8.56	n.d.	n.d.	3.31	5.13	4.41	5.64	n.d.	7.74	n.d.	n.d.	5.07	n.d.
**Eu**	0.64	0.89	1.23	n.d.	n.d.	1.55	n.d.	n.d.	0.81	1.17	0.94	1.13	n.d.	1.54	n.d.	n.d.	1.06	n.d.
**Gd**	2.14	3.35	5.76	n.d.	n.d.	6.93	n.d.	n.d.	2.73	3.94	4.07	4.4	n.d.	6.14	n.d.	n.d.	4.14	n.d.
**Tb**	0.38	0.55	0.8	n.d.	n.d.	1.03	n.d.	n.d.	0.39	0.57	0.6	0.61	n.d.	0.88	n.d.	n.d.	0.61	n.d.
**Dy**	2.37	3.07	4.82	n.d.	n.d.	5.33	n.d.	n.d.	2.13	3.27	3.5	3.55	n.d.	4.68	n.d.	n.d.	3.39	n.d.
**Ho**	0.47	0.62	0.91	n.d.	n.d.	1	n.d.	n.d.	0.42	0.61	0.72	0.69	n.d.	0.82	n.d.	n.d.	0.67	n.d.
**Er**	1.52	1.71	2.85	n.d.	n.d.	2.69	n.d.	n.d.	1.2	1.89	1.93	1.82	n.d.	2.32	n.d.	n.d.	1.96	n.d.
**Tm**	0.24	0.26	0.44	n.d.	n.d.	0.41	n.d.	n.d.	0.18	0.3	0.3	0.29	n.d.	0.33	n.d.	n.d.	0.29	n.d.
**Yb**	1.52	1.65	2.82	n.d.	n.d.	2.52	n.d.	n.d.	1.14	2.13	2.16	1.91	n.d.	2.32	n.d.	n.d.	1.96	n.d.
**Lu**	0.25	0.24	0.46	n.d.	n.d.	0.38	n.d.	n.d.	0.17	0.32	0.32	0.27	n.d.	0.31	n.d.	n.d.	0.32	n.d.
**Chem.Lit**	Psam.	CaO++	Rest--	Rest++	Psam.	Pelite	Psam.	Psam.	CaO++	Pelite	CaO++	Rest++	Psam.	Psam.	Psam.	Rest++	Rest--	Rest--
**Zr/Ti**	224.84	329.7	384.87	250.9	260.37	370.69	248.93	422.41	407.74	317.66	264.56	438.43	264.86	332.4	320.75	285.36	269.51	233.33
**Nb/Y**	0.71	0.34	0.23	0.54	0.78	0.44	0.6	0.53	0.57	0.5	0.32	0.53	0.46	0.43	0.54	0.39	0.45	0.57
**Th/Sc**	1.9	1.48	0.99	1.19	1.04	0.99	2.12	1.75	1.84	1.03	1.3	1.92	0.74	1.94	1.09	1.79	0.82	0.37
**Ti/Nb**	217	340	351	252	204	473	188	208	251	423	406	315	241	327	265	241	453	244
**CIA**	60.4	58.5	68.8	67.5	65.5	63.5	66.3	61.7	62.2	58.6	55.9	58.7	70.9	68	73.5	66.9	62.1	92.7
**PIA**	72.1	64.9	78.5	76.9	75.3	66.4	79.3	66.6	77.7	61.6	59.7	62.8	80.6	71.9	82.4	87.2	64	94.5
**CIW**	82.1	74.5	82.9	81.8	81.3	69.6	85.2	72.4	86.4	67.1	69.6	69.9	84.2	74.6	85.2	92	66.7	94.6
Eu/Eu*	0.83	0.73	0.6	n.d.	n.d.	0.62	n.d.	n.d.	0.82	0.8	0.68	0.69	n.d.	0.68	n.d.	n.d.	0.71	n.d.
**REE**	9.11	11.26	9.7	n.d.	n.d.	16.01	n.d.	n.d.	16	12.88	12.2	19	n.d.	18.93	n.d.	n.d.	11.27	n.d.
**LREE**	5	4.22	3.74	n.d.	n.d.	4.39	n.d.	n.d.	5.13	4.98	5.57	5.99	n.d.	5.29	n.d.	n.d.	4.06	n.d.
**HREE**	1.14	1.65	1.66	n.d.	n.d.	2.23	n.d.	n.d.	1.94	1.5	1.53	1.87	n.d.	2.14	n.d.	n.d.	1.71	n.d.
**Σ REE**	93.56	122.81	196.68	n.d.	n.d.	272.62	n.d.	n.d.	116.98	175.06	167.99	218.63	n.d.	281.22	n.d.	n.d.	151.7	n.d.

Note: Oxides and LOI in %, other elements in ppm. X is mean value for each group of sandstones; SD is standard derivation for that mean. Abbreviations: n.d.: not detected; CaCO* = maximum CaO in Carbonates recalculated from CO_2_; Chem.Lit: Chemical lithology [7] (s. Fig. 7); CaO++: CaO enriched samples; Psam.: Psammite classified samples; Rest++: enriched in SiO_2_ and Al_2_O_3_; Rest--: impoverished in SiO_2_ and Al_2_O_3_;. Eu/Eu* = Eu_N_/(Sm_N_xGd_N_)^0.5^ Samples are not LOI-free recalculated. Samples marked with (*) analyzed by ACME Laboratories, Canada.

**Table 12:** Geochemical parameters of samples from Section III (CaO* = recalculated CaO free of CaO in Carbonates).

**Sample**	**ET-115**	ET-116*	ET-117*	**ET-119**	ET-121*	**ET-125**	ET-126*	**ET-127**	**ET-145**	ET-153*
**SiO**_**2**_	67.5	45.72	66.56	70.71	78.21	68.01	67.55	71.39	66.14	68.8
**TiO**_**2**_	0.61	0.29	0.74	0.57	0.19	0.63	0.55	0.48	0.78	1.3
**Al**_**2**_**O**_**3**_	14.66	9.39	14.75	13.89	12.78	14.87	15.61	14.61	14.48	11.29
**Fe**_**2**_**O**_**3**_	4.44	2.69	5.95	4.89	0.58	6.64	5.27	4.6	7.78	2.68
**MnO**	0.05	0.3	0.06	0.05	0	0.05	0.03	0.03	0.09	0.08
**MgO**	1.12	0.5	0.93	1.2	0.43	1.25	1.26	1.1	1.75	1.16
**CaO**	1.78	19.1	0.87	0.52	0.34	0.44	0.38	0.25	0.93	3.58
CaO*	1.18	18.92	0.02	n.d.	n.d.	0.22	0.01	0.02	0.17	2.11
**Na**_**2**_**O**	1.39	1.95	2.99	0.99	0.35	1.38	1.27	1.43	1.53	2.07
**K**_**2**_**O**	3.88	2.79	2.73	3.96	3.4	2.83	3.66	2.98	2.86	3.34
**P**_**2**_**O**_**5**_	0.08	0.15	0.21	0.34	0.03	0.16	0.05	0.06	0.27	0.2
**LOI**	4.65	17	3.9	2.88	3.6	3.82	4.2	3.12	3.46	5.2
**CO2**	1.73	16.19	0.04	n.d.	n.d.	0.86	0.05	0.13	0.48	2.4
**CaCO**_3_*	2.11	33.77	0.04	n.d.	n.d.	0.39	0.02	0.04	0.3	3.77
**Cr**	29	20	41	23	0	23	34	16	46	41
**Ni**	1	7	14	3	0	4	13	0	17	10
**Co**	17	4	15	14	0	13	7	12	25	5
**Sc**	10	5	10	10	1	14	11	12	14	9
**V**	64	37	105	59	20	56	79	47	85	56
**Pb**	62	7	11	43	4	40	26	47	39	17
**Zn**	74	33	103	80	2	122	85	78	115	59
**Rb**	167	88	112	166	144	125	156	180	116	116
**Ba**	924	716	642	1011	540	476	987	536	800	1430
**Sr**	202	493	198	113	59	88	86	92	128	316
**Ga**	20	8	19	21	12	22	19	22	22	12
**Ta**	0.87	0.5	1.1	1.2	0.6	0.98	1	1.13	1.04	1.8
**Nb**	13.9	6.2	15.7	13.4	10.4	13.1	11.6	14.4	16.9	26.8
**Hf**	11.15	3.1	14.4	9.28	3.4	9.38	4.1	9.63	10.95	10.6
**Zr**	220	104	596	135	101	147	143	136	218	419
**Y**	26	10	27	27	7	25	22	28	30	22
**Th**	17.1	7.2	22.2	16.05	21.1	12.85	15.7	18.15	14.05	32.9
**U**	4.47	1.7	5	2.49	2.1	2.77	5.3	3.76	2.86	3.8
**La**	n.d.	23.6	72.5	n.d.	28.9	n.d.	42.5	7.15	n.d.	129.5
**Ce**	n.d.	46.6	150	n.d.	52	n.d.	85.9	11.74	n.d.	243.1
**Pr**	n.d.	5.08	16.03	n.d.	4.52	n.d.	9.64	n.d.	n.d.	24.01
**Nd**	n.d.	15.9	57.5	n.d.	13.6	n.d.	37.3	8.87	n.d.	79.1
**Sm**	n.d.	2.78	9.79	n.d.	1.71	n.d.	6.72	1.36	n.d.	11.28
**Eu**	n.d.	0.59	1.56	n.d.	0.37	n.d.	1.37	0.21	n.d.	1.67
**Gd**	n.d.	2.41	7.05	n.d.	1.5	n.d.	5.39	1.9	n.d.	7.69
**Tb**	n.d.	0.37	1.04	n.d.	0.25	n.d.	0.79	0.69	n.d.	0.98
**Dy**	n.d.	1.86	5.33	n.d.	1.27	n.d.	4.31	1.71	n.d.	4.8
**Ho**	n.d.	0.3	1	n.d.	0.3	n.d.	0.82	n.d.	n.d.	0.76
**Er**	n.d.	0.93	2.84	n.d.	0.88	n.d.	2.56	0.66	n.d.	2.35
**Tm**	n.d.	0.15	0.45	n.d.	0.17	n.d.	0.36	n.d.	n.d.	0.36
**Yb**	n.d.	1.03	3.03	n.d.	1.29	n.d.	2.5	1.04	n.d.	2.24
**Lu**	n.d.	0.17	0.47	n.d.	0.19	n.d.	0.38	0.14	n.d.	0.38
**Chem.Lit**	Pelite	CaO++	Pelite	Psam.	Psam.	Rest++	Pelite	Rest++	Pelite	Psam.
**Zr/Ti**	360.65	360	806.08	236.84	532.1	233.33	260.54	283.33	279.48	322.46
**Nb/Y**	0.53	0.59	0.56	0.49	1.36	0.52	0.52	0.51	0.56	1.19
**Th/Sc**	1.71	1.44	2.21	1.6	21.1	0.91	1.42	1.51	1	3.65
**Ti/Nb**	263	280	283	255	110	288	284	200	277	291
**CIA**	66.4	60.1	62.2	69.4	72.7	73.4	70.2	71.3	69.3	54.9
**PIA**	76.5	66.5	66.2	83.9	89	83.5	81.4	81.2	77.4	57.6
**CIW**	82	74.5	71.1	88.3	91.9	86.5	85.5	84.7	81.4	66.7
Eu/Eu*	n.d.	0.7	0.57	n.d.	0.71	n.d.	0.7	0.4	n.d.	0.55
**REE**	n.d.	15.48	16.17	n.d.	15.14	n.d.	11.49	4.65	n.d.	39.07
**LREE**	n.d.	5.34	4.66	n.d.	10.64	n.d.	3.98	3.31	n.d.	7.23
**HREE**	n.d.	1.9	1.89	n.d.	0.94	n.d.	1.75	1.48	n.d.	2.78
**Σ REE**	n.d.	101.77	328.59	n.d.	106.95	n.d.	200.54	35.47	n.d.	508.22

Note: Oxides and LOI in %, other elements in ppm. X is mean value for each group of sandstones; SD is standard derivation for that mean. Abbreviations: n.d.: not detected; CaCO* = maximum CaO in Carbonates recalculated from CO_2_; Chem.Lit: Chemical lithology [7] (s. Fig. 7); CaO++: CaO enriched samples; Psam.: Psammite classified samples; Rest++: enriched in SiO_2_ and Al_2_O_3_; Rest--: impoverished in SiO_2_ and Al_2_O_3_;. Eu/Eu* = Eu_N_/(Sm_N_xGd_N_)^0.5^ Samples are not LOI-free recalculated. Samples marked with (*) analyzed by ACME Laboratories, Canada.

**Table 13:** Geochemical parameters of samples from Section IV (CaO* = recalculated CaO free of CaO in Carbonates).

**Sample**	ET-160*	**ET-161**	**ET-164**	ET-167*	**ET-168**	ET-174*	**ET-176**	**ET-178**
**SiO**_**2**_	75.23	75.46	73.09	45.08	50.01	73.36	15.82	70.72
**TiO**_**2**_	0.37	0.48	0.57	0.27	0.22	0.36	0.11	0.55
**Al**_**2**_**O**_**3**_	12.74	12.82	12.96	8.38	5.81	13.67	3.81	11.72
**Fe**_**2**_**O**_**3**_	0.63	1.03	3.66	1.8	2.31	1.65	1.99	5.1
**MnO**	n.d.	0.02	0.06	0.46	0.61	0.02	0.61	0.06
**MgO**	0.23	0.34	1.14	0.6	2.71	0.8	0.35	2.61
**CaO**	0.15	0.16	0.82	21.06	21.02	0.85	49.88	1.11
CaO*	n.d.	n.d.	n.d.	18.94	17.74	0.08	41.59	0.39
**Na**_**2**_**O**	0.28	0.35	1.43	2.68	0.37	3.88	n.d.	0.68
**K**_**2**_**O**	8.34	8.04	3.9	0.94	0.64	2.06	1.1	3.01
**P**_**2**_**O**_**5**_	0.02	0.08	0.15	0.1	0.03	0.04	n.d.	0.15
**LOI**	1.8	1.22	2.23	18.5	18.68	3.1	34.47	4.24
**CO2**	n.d.	n.d.	n.d.	15.45	16.42	0.15	32.96	1.3
**CaCO**_3_*	n.d.	n.d.	n.d.	33.8	31.66	0.14	74.23	0.7
**Cr**	n.d.	22	16	13	12	20	12	38
**Ni**	1	11	0	7	8	10	14	13
**Co**	0	6	10	4	40	2	14	13
**Sc**	5	5	11	5	13	5	9	9
**V**	36	54	70	47	48	42	58	55
**Pb**	4	35	38	10	19	18	37	45
**Zn**	3	42	61	15	27	26	31	59
**Rb**	316	339	168	45	55	78	92	185
**Ba**	1473	1682	851	387	287	335	559	312
**Sr**	95	80	147	308	449	371	843	71
**Ga**	11	14	20	6	12	12	10	22
**Ta**	0.6	1.69	0.87	0.3	0.97	0.8	0.4	1.63
**Nb**	12.3	11.4	12.4	4.6	8.7	9.1	5.8	26.4
**Hf**	3.7	3.94	12.25	2.5	8.75	8.5	4.88	13.05
**Zr**	128	158	192	100	162	322	209	186
**Y**	19	30	25	31	56	18	93	39
**Th**	12.8	12.85	13.25	5.4	7.47	15.5	6.96	25.7
**U**	3.4	4.42	2.91	1.3	1.97	2.1	1.23	3.74
**La**	36.9	n.d.	31.9	31.5	52.46	48.9	51.73	n.d.
**Ce**	61.3	n.d.	66.18	46.9	78.59	88.9	129.2	n.d.
**Pr**	6.36	n.d.	9.25	6.89	n.d.	9.56	16.91	n.d.
**Nd**	21	n.d.	36.75	27.9	37.22	33.5	64.66	n.d.
**Sm**	3.63	n.d.	8.21	6.14	10.65	5.51	12.25	n.d.
**Eu**	0.61	n.d.	1.84	1.33	1.4	0.89	3.17	n.d.
**Gd**	3.31	n.d.	8.58	5.72	10.3	4.71	9.99	n.d.
**Tb**	0.48	n.d.	1.31	0.86	1.13	0.61	1.31	n.d.
**Dy**	2.89	n.d.	6.86	4.54	7.01	3.19	6.06	n.d.
**Ho**	0.67	n.d.	n.d.	0.91	n.d.	0.63	n.d.	n.d.
**Er**	1.88	n.d.	3.72	2.32	1.14	1.7	2.3	n.d.
**Tm**	0.34	n.d.	n.d.	0.33	n.d.	0.28	n.d.	n.d.
**Yb**	2.25	n.d.	3.81	2.2	3.03	1.9	2.42	n.d.
**Lu**	0.37	n.d.	0.48	0.31	0.35	0.3	0.32	n.d.
**Chem.Lit**	Psam.	Psam.	Psam.	CaO++	CaO++	Psam.	CaO++	Psam.
**Zr/Ti**	346.48	329.16	336.84	372.59	736.36	895.27	1900	338.18
**Nb/Y**	0.64	0.38	0.49	0.14	0.15	0.48	0.06	0.67
**Th/Sc**	2.56	2.57	1.2	1.08	0.57	3.1	0.77	2.85
**Ti/Nb**	180	252	276	352	152	237	114	125
**CIA**	56.7	57.7	62.6	48	44.7	57.9	19	68.6
**PIA**	84.2	85.4	71.3	47.8	44	59.8	14.8	80.1
**CIW**	94.8	94.8	78.7	51	47.2	64	20.2	84.8
Eu/Eu*	0.54	n.d.	0.67	0.69	0.41	0.53	0.88	n.d.
**REE**	11.08	n.d.	5.66	9.68	11.7	17.39	14.44	n.d.
**LREE**	6.4	n.d.	2.45	3.23	3.1	5.59	2.66	n.d.
**HREE**	1.19	n.d.	1.83	2.11	2.76	2.01	3.35	n.d.
**Σ REE**	141.99	n.d.	178.89	137.85	203.28	200.58	300.32	n.d.

Note: Oxides and LOI in %, other elements in ppm. X is mean value for each group of sandstones; SD is standard derivation for that mean. Abbreviations: n.d.: not detected; CaCO* = maximum CaO in Carbonates recalculated from CO_2_; Chem.Lit: Chemical lithology [7] (s. Fig. 7); CaO++: CaO enriched samples; Psam.: Psammite classified samples; Rest++: enriched in SiO_2_ and Al_2_O_3_; Rest--: impoverished in SiO_2_ and Al_2_O_3_;. Eu/Eu* = Eu_N_/(Sm_N_xGd_N_)^0.5^ Samples are not LOI-free recalculated.

**Table 14:** Geochemical parameters of samples from Section V (CaO* = recalculated CaO free of CaO in Carbonates).

**Sample**	ET-180*	**ET-181**	ET-185*
**SiO**_**2**_	74.69	76.5	71.01
**TiO**_**2**_	0.28	0.3	0.3
**Al**_**2**_**O**_**3**_	13.35	11.1	13.26
**Fe**_**2**_**O**_**3**_	1.78	2.47	1.64
**MnO**	0.01	0.02	0.05
**MgO**	0.25	0.34	0.53
**CaO**	0.18	0.23	1.86
CaO*	0.01	n.d.	1.06
**Na**_**2**_**O**	4.88	1.63	1.55
**K**_**2**_**O**	3.01	5.83	5.08
**P**_**2**_**O**_**5**_	0.05	0.16	0.06
**LOI**	1.4	1.38	4.5
**CO2**	0.02	n.d.	1.16
**CaCO**_3_*	0.02	n.d.	1.89
**Cr**	20	14	0
**Ni**	6	9	7
**Co**	8	11	2
**Sc**	4	4	4
**V**	35	105	27
**Pb**	12	123	21
**Zn**	30	13	26
**Rb**	88	230	153
**Ba**	775	1401	1113
**Sr**	200	104	153
**Ga**	12	11	14
**Ta**	0.4	2.33	1
**Nb**	8.5	15.2	12.8
**Hf**	4	5.47	5.1
**Zr**	141	200	197
**Y**	16	22	21
**Th**	9	15.25	13.6
**U**	2.3	4.95	2.3
**La**	30.2	n.d.	48.2
**Ce**	58.9	n.d.	89.8
**Pr**	6.48	n.d.	9.92
**Nd**	24.4	n.d.	35.1
**Sm**	4.27	n.d.	5.33
**Eu**	0.77	n.d.	0.97
**Gd**	3.56	n.d.	4.95
**Tb**	0.51	n.d.	0.65
**Dy**	3.28	n.d.	3.72
**Ho**	0.65	n.d.	0.72
**Er**	1.83	n.d.	2.03
**Tm**	0.26	n.d.	0.35
**Yb**	1.8	n.d.	2.31
**Lu**	0.28	n.d.	0.35
**Chem.Lit**	Psam.	Psam.	Psam.
**Zr/Ti**	506.78	666.66	659.33
**Nb/Y**	0.52	0.69	0.58
**Th/Sc**	2.25	3.81	3.4
**Ti/Nb**	197	118	141
**CIA**	53.8	55	58.6
**PIA**	55.1	63.5	66.7
**CIW**	61.9	80.1	77.4
Eu/Eu*	0.6	n.d.	0.58
**REE**	11.34	n.d.	14.1
**LREE**	4.45	n.d.	5.69
**HREE**	1.6	n.d.	1.74
**Σ REE**	137.19	n.d.	204.4

Note: Oxides and LOI in %, other elements in ppm. X is mean value for each group of sandstones; SD is standard derivation for that mean. Abbreviations: n.d.: not detected; CaCO* = maximum CaO in Carbonates recalculated from CO_2_; Chem.Lit: Chemical lithology [7] (s. Fig. 7); CaO++: CaO enriched samples; Psam.: Psammite classified samples; Rest++: enriched in SiO_2_ and Al_2_O_3_; Rest--: impoverished in SiO_2_ and Al_2_O_3_;. Eu/Eu* = Eu_N_/(Sm_N_xGd_N_)^0.5^ Samples are not LOI-free recalculated.

**Table 15:** Geochemical parameters of samples from El Tranquilo igneous rocks (CaO* = recalculated CaO free of CaO in Carbonates).

**Sample**	**ET-38**	**ET-41**	**ET-42**	ET-43*	**ET-60**	**ET-64**	**ET-67**	ET-91*	**ET-99**	**ET-109**	ET-113*	ET-134*	ET-135*	**ET-140**	ET-141*	**ET-151**	ET-175*
**SiO**_**2**_	80.92	51.8	57.11	52.19	50.48	69.74	72.82	69.22	73.91	51.01	52.75	50.12	48.21	52.72	45.79	74.89	15.99
**TiO**_**2**_	0.25	1.25	1.89	1.46	1.45	0.56	0.58	0.34	0.48	1.01	0.75	0.7	0.79	0.76	0.79	0.34	0.27
**Al**_**2**_**O**_**3**_	12.1	16.21	13.38	16.9	16.5	14.45	13.4	14.14	12.81	16.08	15.15	14.56	16.06	14.87	16.15	10.95	9.61
**Fe**_**2**_**O**_**3**_	0.62	9.82	10.27	9.97	9.58	2.58	3.73	2.52	4.66	8.53	9.99	9.66	9.16	9.79	9.23	1.81	67.38
**MnO**	0.01	0.14	0.17	0.14	0.23	0.03	0.04	0.05	0.02	0.14	0.18	0.17	0.14	0.15	0.14	0.06	0.48
**MgO**	0.34	5.47	2.77	3.74	4.19	0.59	0.91	1.03	1.68	5.95	5.41	5.13	3.24	4.29	3.44	0.82	0.06
**CaO**	0.02	8.98	5.25	8.24	8.76	1.13	0.55	2.36	0.37	3.97	9.16	9.65	7.27	9.07	8.4	3.07	0.34
CaO*	n.d.	n.d.	n.d.	0.05	1.46	0.15	n.d.	1.15	0.02	1.78	1.18	2.97	4.66	2.7	5.13	0.96	0.02
**Na**_**2**_**O**	n.d.	2.56	3	3.19	2.09	3.84	2.05	2.59	1.17	3.51	1.66	1.2	2.65	0.9	2.36	2.21	0.11
**K**_**2**_**O**	2.77	1.77	2.91	1.77	1.53	3.81	3.4	2.25	1.76	2.69	1.43	1.23	1.96	1.33	2	1.89	0.52
**P**_**2**_**O**_**5**_	0.05	0.42	1.03	0.39	0.52	0.12	0.15	0.06	0.11	0.38	0.1	0.08	0.11	0.1	0.11	0.1	0.15
**LOI**	3	1.59	2.25	1.7	4.82	3.2	2.4	5.3	3.12	7.12	3.1	7.2	10.2	6.35	11.3	3.98	4.8
**CO2**	n.d.	n.d.	n.d.	0.06	1.91	0.21	n.d.	1.45	0.13	4.32	1.69	4.05	5.92	3.51	6.32	1.03	0.02
**CaCO**_3_*	n.d.	n.d.	n.d.	0.09	2.61	0.27	n.d.	2.05	0.04	3.18	2.11	5.3	8.32	4.82	9.16	1.71	0.04
**Cr**	11	121	5	34	141	16	30	13	19	161	136	136	136	153	143	19	27
**Ni**	6	56	0	27	73	0	14	7	13	50	18	55	66	74	67	11	28
**Co**	9	42	35	23	45	16	18	5	19	32	38	35	36	50	41	19	24
**Sc**	10	30	26	29	28	10	9	7	11	23	36	34	38	43	38	7	25
**V**	24	220	198	312	250	67	91	43	67	179	198	192	210	225	207	46	786
**Pb**	37	10	55	6	47	68	56	20	26	42	4	7	8	8	9	32	76
**Zn**	13	85	122	45	98	59	50	45	49	58	47	58	80	76	81	55	55
**Rb**	154	43	67	44	34	100	128	100	75	90	42	41	68	46	73	84	24
**Ba**	246	683	982	664	658	671	1564	757	872	1105	753	1010	325	1290	282	407	218
**Sr**	41	572	424	606	573	247	411	209	188	451	175	269	318	264	394	250	85
**Ga**	14	19	20	19	20	16	16	14	14	17	18	14	19	16	19	12	14
**Ta**	1.79	1.15	1.51	0.3	1.64	1.49	1.3	0.4	1.22	1.25	0.5	0.4	0.7	0.17	0.6	1.44	0.2
**Nb**	16.6	13.2	24.4	10.4	14.8	10.4	14.7	9.1	12.2	15.5	6.3	5	5.9	7.6	6.2	12.9	6.2
**Hf**	8.34	4.77	14.5	5.3	6.43	10.6	5.15	4.4	6.03	6.88	4.3	3.5	4.5	3.8	4.3	5.24	3.4
**Zr**	240	211	376	221	253	172	206	145	176	127	155	138	154	152	162	160	185
**Y**	35	32	60	29	38	24	14	23	19	24	28	28	25	32	25	17	14
**Th**	15.45	5.32	6.28	3.4	4.22	10.58	14.9	12.4	12.3	6.11	5.5	6.6	6.8	8.68	6.8	13.95	7.6
**U**	1.88	1.26	1.76	0.8	1.45	3.59	3.89	2.5	2.36	2.29	1.8	1.6	2	1.04	1.9	1.51	12.9
**La**	n.d.	n.d.	79.31	30.4	n.d.	n.d.	n.d.	41.1	n.d.	34.32	18.5	19.4	17.4	n.d.	20.6	n.d.	8.8
**Ce**	n.d.	n.d.	115.1	68.8	n.d.	n.d.	n.d.	81.6	n.d.	42.37	42.8	38.6	39.7	n.d.	43.9	n.d.	78.5
**Pr**	n.d.	n.d.	15.46	8.52	n.d.	n.d.	n.d.	8.57	n.d.	1.17	4.94	4.5	4.52	n.d.	5.18	n.d.	2.81
**Nd**	n.d.	n.d.	50.13	36.4	n.d.	n.d.	n.d.	31.5	n.d.	64.01	20.5	19.2	19	n.d.	21	n.d.	12.5
**Sm**	n.d.	n.d.	8.22	7.11	n.d.	n.d.	n.d.	5.49	n.d.	2.18	4.31	3.92	4.29	n.d.	4.68	n.d.	3.59
**Eu**	n.d.	n.d.	1.89	1.83	n.d.	n.d.	n.d.	1.03	n.d.	1.24	1.18	1.09	1.13	n.d.	1.14	n.d.	0.88
**Gd**	n.d.	n.d.	6.09	6.66	n.d.	n.d.	n.d.	4.88	n.d.	1.69	4.71	4.11	4.12	n.d.	4.8	n.d.	4.1
**Tb**	n.d.	n.d.	0.54	0.95	n.d.	n.d.	n.d.	0.73	n.d.	n.d.	0.81	0.73	0.74	n.d.	0.8	n.d.	0.75
**Dy**	n.d.	n.d.	2.84	5.1	n.d.	n.d.	n.d.	3.83	n.d.	0.01	5.39	4.71	5.1	n.d.	5.26	n.d.	4.06
**Ho**	n.d.	n.d.	n.d.	1.12	n.d.	n.d.	n.d.	0.81	n.d.	n.d.	1.02	0.96	1.07	n.d.	1.07	n.d.	0.79
**Er**	n.d.	n.d.	0.99	3.05	n.d.	n.d.	n.d.	2.3	n.d.	0.12	3.02	2.76	3.01	n.d.	3.13	n.d.	2.2
**Tm**	n.d.	n.d.	n.d.	0.44	n.d.	n.d.	n.d.	0.33	n.d.	n.d.	0.49	0.43	0.46	n.d.	0.46	n.d.	0.37
**Yb**	n.d.	n.d.	0.91	2.95	n.d.	n.d.	n.d.	2.25	n.d.	n.d.	3.18	2.81	3.18	n.d.	2.94	n.d.	2.53
**Lu**	n.d.	n.d.	0.12	0.5	n.d.	n.d.	n.d.	0.37	n.d.	0.03	0.5	0.45	0.44	n.d.	0.43	n.d.	0.4
**Chem.Lit**	Psam.	CaO++	CaO++	CaO++	CaO++	Rest++	Psam.	Rest++	Psam.	Pelite	CaO++	CaO++	CaO++	CaO++	CaO++	Psam.	Rest--
**Zr/Ti**	960	168.8	198.94	151.5	174.48	307.14	355.17	426.76	366.66	125.74	207.33	197.14	195.94	200	206.07	470.58	686.29
**Nb/Y**	0.47	0.41	0.4	0.35	0.38	0.43	1.05	0.39	0.64	0.64	0.22	0.17	0.22	0.23	0.24	0.75	0.41
**Th/Sc**	1.54	0.17	0.24	0.11	0.15	1.05	1.65	1.77	1.11	0.26	0.15	0.19	0.17	0.2	0.17	1.99	0.3
**Ti/Nb**	90	568	464	842	587	323	237	224	236	391	714	839	803	600	764	158	261
**CIA**	80.1	42.9	46.5	44.3	48.9	54.7	63.4	61.7	75.2	57.6	44.9	48.8	59.4	51	57.9	54.1	90.6
**PIA**	100	42.1	45.5	43.7	48.8	56.8	70.6	64.9	82.4	59.6	44.4	48.7	61.1	51.1	59.3	55.1	95.4
**CIW**	100	45.2	52.2	46.7	51.4	64.8	76.8	69.1	84.6	64.3	47.1	51.1	64.4	53.7	62.7	60.2	95.7
Eu/Eu*	n.d.	n.d.	0.82	0.81	n.d.	n.d.	n.d.	0.61	n.d.	1.97	0.8	0.83	0.82	n.d.	0.74	n.d.	0.7
**REE**	n.d.	n.d.	58.89	6.96	n.d.	n.d.	n.d.	12.34	n.d.	n.d.	3.93	4.67	3.7	n.d.	4.73	n.d.	2.35
**LREE**	n.d.	n.d.	6.07	2.69	n.d.	n.d.	n.d.	4.71	n.d.	9.91	2.7	3.12	2.55	n.d.	2.77	n.d.	1.54
**HREE**	n.d.	n.d.	5.42	1.83	n.d.	n.d.	n.d.	1.76	n.d.	n.d.	1.2	1.19	1.05	n.d.	1.32	n.d.	1.31
**Σ REE**	n.d.	n.d	281.6	173.83	n.d.	n.d.	n.d	184.79	0	147.14	111.35	103.67	104.16	n.d.	115.39	n.d.	122.28

Note: Oxides and LOI in %, other elements in ppm. X is mean value for each group of sandstones; SD is standard derivation for that mean. Abbreviations: n.d.: not detected; CaCO* = maximum CaO in Carbonates recalculated from CO_2_; Chem.Lit: Chemical lithology [7] (s. Fig. 7); CaO++: CaO enriched samples; Psam.: Psammite classified samples; Rest++: enriched in SiO_2_ and Al_2_O_3_; Rest--: impoverished in SiO_2_ and Al_2_O_3_;. Eu/Eu* = Eu_N_/(Sm_N_xGd_N_)^0.5^ Samples are not LOI-free recalculated.

**Table 16:** Simple statistics of the selected geochemical parameters of the El Tranquilo Group, Cañadón Largo Formation, and Laguna Colorada Formation.

		SiO_2_/AL_2_O_3_	K_2_O/Na_2_O	CIA	Ti/Nb	SiO_2_/K_2_O	Th/Sc	Zr/Sc
**El Tranquilo group**	**Mean**	5.26	3.35	61	288	23.38	2.01	21.91
	−**95%**	4.99	2.11	59	254	19.32	1.5	18.03
	**+95%**	5.53	4.59	63	322	25.44	2.52	25.8
	−**99%**	4.9	1.7	58	243	18.30	1.33	16.76
	**+99%**	5.62	5	64	333	26.45	2.68	27.06
**Cañadón Largo Fm.**	**Mean**	5.43	2.7	63	252	21.25	2.31	22.87
	−**95%**	5.16	1.99	62	232	18.87	1.5	17.22
	**+95%**	5.69	3.41	65	273	23.63	3.11	28.53
	−**99%**	5.07	1.76	61	225	18.05	1.23	15.33
	**+99%**	5.78	3.64	66	280	24.42	3.38	30.42
**Laguna Colorada Fm. (Section IV)**	**Mean**	5.87	8.93	52	211	29.59	1.84	26.95
	**-95%**	4.82	-2.32	39	142	9.74	0.98	13.43
	**+95%**	6.91	20.17	65	280	49.45	2.7	40.47
	−**99%**	4.31	-8.11	33	109	0.21	0.57	6.94
	**+99%**	7.42	25.96	71	313	58.98	3.11	46.96

**Table 17:** Simple statistics of selected Trace and rare earth elements (REEs) of the El Tranquilo Group, Cañadón Largo Formation, and Laguna Colorada Formation.

		**Rb (ppm)**	**Ba (ppm)**	**Sr (ppm)**	**La (ppm)**	**Ce (ppm)**	**Pr (ppm)**	**Nd (ppm)**	**Sm (ppm)**	**Eu (ppm)**	**Gd (ppm)**	**Tb (ppm)**	**Dy (ppm)**	**Ho (ppm)**	**Er (ppm)**	**Tm (ppm)**	**Yb (ppm)**	**Lu (ppm)**	Eu/Eu*	**La**_**N**_**/Yb**_**N**_	**La**_**N**_**/Sm**_**N**_	**Gd**_**N**_**/Yb**_**N**_	**Σ REE (ppm)**
**El Tranquilo anticline**	**Mean**	134	1030	224	41.02	79.93	8.80	33.19	5.75	1.20	4.99	0.73	3.96	0.73	2.07	0.32	2.22	0.33	0.72	14.07	4.70	1.91	184.58
	−**95%**	148	1261	255	47.98	92.95	10.27	38.23	6.60	1.37	5.70	0.82	4.47	0.81	2.34	0.36	2.47	0.37	0.79	17.17	5.26	2.16	211.82
	**+95%**	153	1336	265	50.33	97.35	10.76	39.93	6.88	1.42	5.94	0.85	4.64	0.84	2.43	0.37	2.56	0.39	0.81	18.22	5.45	2.24	221.01
	−**99%**	120	798	194	34.06	66.91	7.34	28.15	4.91	1.03	4.28	0.65	3.44	0.66	1.80	0.29	1.96	0.29	0.64	10.97	4.14	1.67	157.34
	**+99%**	115	723	184	31.71	62.51	6.85	26.45	4.62	0.98	4.04	0.62	3.27	0.63	1.70	0.28	1.88	0.27	0.62	9.92	3.95	1.59	148.14
**Cañadón Largo Fm.**	**Mean**	147	1153	194	45.79	89.31	9.86	34.22	5.81	1.14	4.80	0.70	3.78	0.68	2.03	0.30	2.07	0.32	0.67	15.21	5.08	1.85	200.30
	−**95%**	162	1522	222	56.91	111.06	12.02	41.89	7.06	1.35	5.80	0.83	4.49	0.78	2.40	0.34	2.44	0.39	0.72	18.28	5.74	2.06	246.05
	**+95%**	166	1645	232	60.91	118.88	12.80	44.64	7.50	1.43	6.16	0.87	4.74	0.81	2.53	0.36	2.57	0.41	0.74	19.38	5.97	2.13	262.48
	−**99%**	133	784	167	34.68	67.55	7.70	26.54	4.57	0.92	3.80	0.58	3.07	0.58	1.65	0.26	1.71	0.26	0.63	12.14	4.43	1.65	154.55
	**+99%**	129	661	157	30.68	59.74	6.92	23.79	4.13	0.84	3.44	0.54	2.82	0.54	1.52	0.24	1.58	0.23	0.61	11.04	4.20	1.57	138.12
**Laguna Colorada Fm. (Section IV)**	**Mean**	159	736	295	42.23	78.51	9.79	36.84	7.73	1.54	7.10	0.95	5.09	0.74	2.18	0.32	2.60	0.36	0.62	11.66	3.91	2.21	193.82
	−**95%**	255	1199	516	52.62	108.65	15.03	52.52	11.16	2.49	10.17	1.32	7.00	1.11	3.09	0.40	3.34	0.42	0.79	15.88	5.65	3.00	255.94
	**+95%**	302	1421	622	58.52	125.79	18.48	61.43	13.11	3.03	11.92	1.54	8.08	1.60	3.61	0.50	3.75	0.46	0.89	18.28	6.64	3.45	291.26
	−**99%**	63	273	74	31.85	48.37	4.55	21.16	4.30	0.59	4.03	0.58	3.18	0.36	1.26	0.24	1.87	0.29	0.45	7.44	2.16	1.42	131.70
	**+99%**	17	51	-31	25.94	31.23	1.11	12.24	2.35	0.05	2.29	0.36	2.10	-0.13	0.74	0.13	1.45	0.25	0.35	5.04	1.17	0.97	96.38
**Roca Blanca Fm. (Section V)**	**Mean**	153	1123	158	36.58	70.15	7.66	27.95	4.40	0.90	3.84	0.54	3.08	0.59	1.63	0.27	1.71	0.27	0.68	14.87	5.25	1.87	159.56
	−**95%**	206	1609	190	49.79	92.42	10.11	35.77	5.50	1.04	5.04	0.66	3.92	0.78	2.23	0.37	2.46	0.37	0.85	19.32	6.73	2.32	208.66
	**+95%**	230	1822	203	60.83	111.02	12.16	42.30	6.42	1.16	6.04	0.76	4.62	0.94	2.74	0.45	3.10	0.46	0.99	23.03	7.97	2.69	249.69
	−**99%**	99	637	127	23.36	47.88	5.21	20.13	3.30	0.76	2.65	0.42	2.24	0.40	1.03	0.17	0.95	0.16	0.51	10.43	3.76	1.43	110.45
	**+99%**	75	425	113	12.32	29.28	3.16	13.60	2.38	0.64	1.65	0.31	1.53	0.25	0.52	0.09	0.31	0.07	0.37	6.72	2.52	1.05	69.42

**Table 18:** CIA, Ti/Nb ratios, and SiO_2_/K_2_O ratio values of geochemical standards [8] used for comparison in Fig. 10, Fig. 16.

**Standard**	**Rock type**	**CIA**	**Ti/Nb**	**SiO**_**2**_**/K**_**2**_**O**	**Standard**	**Rock type**	**CIA**	**Ti/Nb**	**SiO**_**2**_**/K**_**2**_**O**
**SARM40**	Carbonatite	0.5	30	102	**MK-1**	Granodiorite	48.2	352	16
**JH-1**	Hornblendite	16.1	–	93	**NIM-S**	Syenite	48.6	66	4
**WMG-1**	Gabbro	23.7	–	410	**SKD-1**	Quartz-Diorite	48.6	430	20
**WBG-1**	Gabbro	24.6	–	54	**T-1**	Tonalite	48.6	–	50
**MRG-1**	Gabbro	24.8	1130	217	**JG-3**	Granodiorite	48.8	514	25
**BE-N**	Basalt	25	149	27	**GS-N**	Granite	48.9	194	14
**BR**	Basalt	25.6	159	27	**MO-9**	Anorthosite-Gabbro	48.9	839	107
**SARM48**	Granite	30.1	3	15	**GOG-1**	Gabbro	49	–	898
**MO-3**	Gabbro	31.5	–	184	**JR-3**	Rhyolite	49.1	–	16
**SY-3**	Syenite	31.7	6	14	**MK-4**	Granite	49.1	124	17
**SY-2**	Syenite	32.3	31	13	**MO-13**	Olivine-Basalt	49.2	766	52
**SDG-1a**	Gabbro	34.9	1281	15	**MK-2**	Granodiorite	49.3	252	20
**SDG-2**	Gabbro	35.4	1228	15	**G-B**	Granite	49.3	–	23
**BHVO-1**	Basalt	35.6	855	96	**QLO-1**	Quartz-Latite	49.4	363	18
**BIR-1**	Basalt	36.3	9592	1769	**DVD**	Hornblende Dacite	49.4	420	26
**MO-7**	Orthoklase-Gabbro	36.4	1694	54	**GR**	Granite	49.5	–	14
**GSR-3**	Basalt	36.9	208	19	**BM**	Basalt	49.6	–	247
**JP-1**	Peridotite	37	–	14130	**NIM-G**	Granite	49.9	10	15
**MY-3**	Hornblendite	37.5	–	99	**JG-2**	Granite	50	16	16
**TDB-1**	Diabase	37.8	–	55	**GA**	Granite	50.2	190	17
**W-1**	Diabase	38.4	648	82	**SG-1a**	albitized Granite	50.2	1	17
**GV**	Gabbro	38.4	–	190	**JR-1**	Rhyolite	50.4	39	17
**JB-1**	Basalt	38.7	233	36	**DVT**	biotitic Trachyrhyolite	50.5	7	16
**SARM50**	Dolerite	38.8	516	84	**RGM-1**	Rhyolite	50.6	180	17
**JB-1a**	Basalt	38.9	289	36	**JG-1**	Granodiorite	50.6	124	18
**W-2**	Diabase	39.2	804	83	**GSR-1**	Granite	50.7	43	14
**NIM-N**	Norite	39.8	600	210	**G-2**	Granite	50.7	240	15
**NBS688**	Basalt	40.1	1228	254	**JG-1a**	Granodiorite	50.7	125	18
**JB-2**	Basalt	40.3	8918	126	**PCC-1**	Peridotite	50.7	60	5958
**MB-H**	metabasic Rock	40.8	928	18	**GH**	Granite	50.9	6	15
**BCR-1**	Basalt	40.8	959	32	**G-1**	Granite	51	72	13
**MO-12**	Andesite-Basalt	41.7	689	44	**JR-2**	Rhyolite	51.1	28	17
**MO-2**	Basalt	41.8	–	50	**GSP-1**	Granodiorite	51.7	140	12
**JB-3**	Basalt	41.9	3779	65	**GM**	Granite	51.7	71	15
**GL-O**	Glauconite	42	113	6	**DVR**	albitized Rhyodacite	51.7	213	18
**JGb-1**	Gabbro	42.3	3469	181	**MK-3**	Granite	51.8	126	12
**MO-5**	Gabbro	42.4	4411	47	**SG-2**	Alaskite Granite	52	276	10
**MO-14**	Olivine-Basalt	42.7	883	101	**DTS-1**	Dunite	55.4	14	40409
**MO-1**	Diabase	43.5	885	28	**MA-N**	Granite	56.1	0	20
**MO-8**	Gabbro	43.5	1863	112	**2B**	Granitoid	57.5	1	25
**MO-4**	Gabbro	43.6	–	23	**DVG**	greisenized Granit	57.9	1	21
**DNC-1**	Dolerite	43.6	959	205	**GSR-2**	Andesite	62.2	458	32
**NIM-P**	Pyroxenite	43.8	–	567	**SDC-1**	Mica Shist	63	336	20
**MB**	Monzonite	44.9	–	12	**M I**	Chlorite-Muscovite-Shist	65.1	–	28
**JA-2**	Andesite	44.9	410	31	**JSl-1**	Slate	65.4	–	20
**NS-1**	Nepheline Syenite	45	32	8	**JSl-2**	Slate	66.7	–	20
**DVB**	bipyroxene basaltic Andesite	45	605	27	**MY-1**	Peridotite	66.9	–	1035
**SNS-2**	Nepheline Syenite	45.2	22	9	**MO-15**	porphyric Andesite-Basalt	68	775	26
**WPR-1**	Peridotite	45.3	–	370	**ASK-2**	Shist	69.1	917	10
**MDO-G**	Trachyte	45.7	112	13	**SBO-1**	Shist	69.2	331	15
**ISH-G**	Trachyte	46	87	8	**MY-2**	Dunite	75.1	–	1402
**JGb-2**	Gabbro	46	–	778	**DZE-2**	ultrabasic Rock	77.7	–	3775
**JA-1**	Andesite	46.1	3068	82	**GnA**	Greisen	78.2	1	27
**JA-3**	Andesite	46.2	1359	44	**SARM47**	Serpentinite	79.2	–	1814
**SG-3**	Granite	46.3	92	16	**NIM-D**	Dunite	79.7	–	3896
**STM-1**	Syenite	46.4	3	13	**SSL-2**	Shist	80.1	378	17
**DVA**	Hornblende Hyaloandesite	46.7	1049	68	**SDU-1**	Dunite	93.4	–	3957
**AGV-1**	Andesite	47.3	420	20	**DZE-1**	ultrabasic Rock	96.1	–	3434
**DR-N**	Diorite	47.5	934	31	**SW**	Serpentinite	96.9	–	–
**AC-E**	Granite	47.8	6	15	**SARM44**	Sillimanite Schist	99.1	114	193

For the analysis of 45 samples, the material was crushed and dried to a constant weight at 105 °C. The loss on ignition (LOI) was determined after annealing for 1.5 h at 1,050 °C, than, the material was mixed with one part lithium tetraborate (Li_2_B_4_O_7_) and melted at 1,400 °C in a graphite crucible and poured into platinum pouring bowls. Major and trace element concentrations were determined using a sequentially operating, wavelength-dispersive X-ray fluorescence spectrometer (SIEMENS SRS 303 AS, in the 1990s at the Geological Institute of Ludwig Maximilians University of Munich, Germany) on a volatile-free base (major element concentrations as oxides in weight %, and trace element concentrations in ppm). In this method the measured values for Fe_2_O_3_ are total iron values. Rare earth elements (REEs) were analyzed using atomic emission spectroscopy, with inductively coupled plasma excitation on an ICP-AES (Jobin YVON Model 38 plus). Thirty-five samples were analyzed and pulverized by ICP-ES (for oxides of Ba, Ni, and Sc), and by ICP-MS (for trace elements and REEs) at ACME Laboratories, Vancouver, Canada. This samples are marked with an asterisk (*) in the Table 10, Table 11, Table 12, Table 13, Table 14, Table 15.

**Fig. 7:**
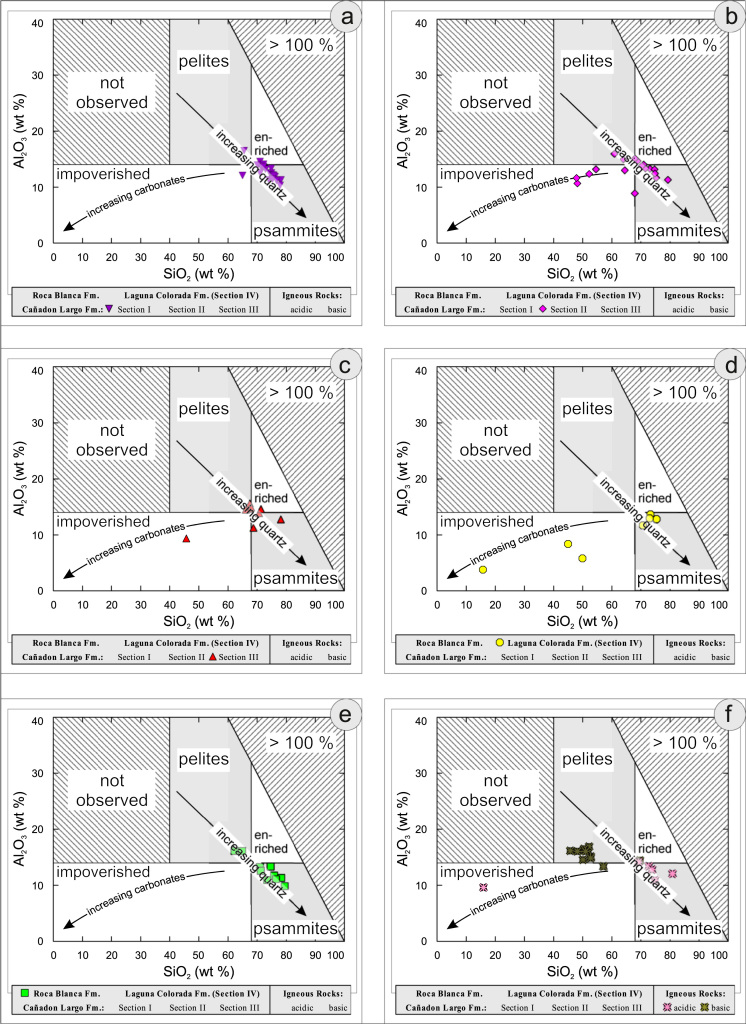
Geochemical sediment classification of El Tranquilo sediments and igneous rocks: SiO_2_–Al_2_O_3_ after [9] (modified).

**Fig. 8:**
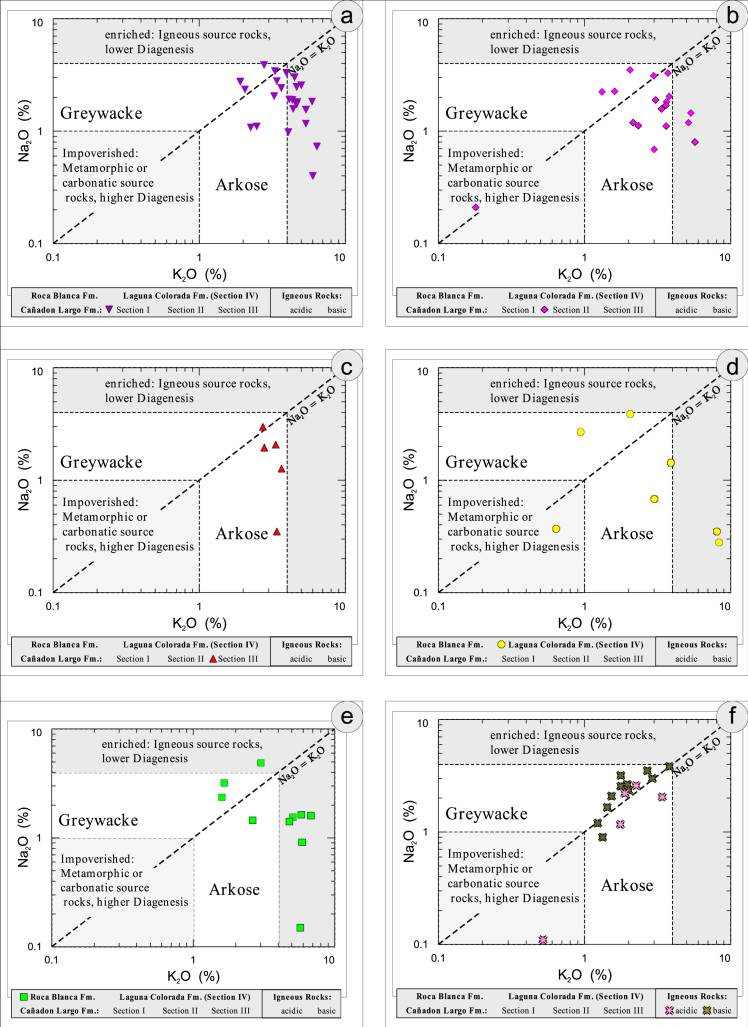
Geochemical sediment classification of El Tranquilo sediments and igneous rocks: K_2_O–Na_2_O after [10] (modified).

**Fig. 9:**
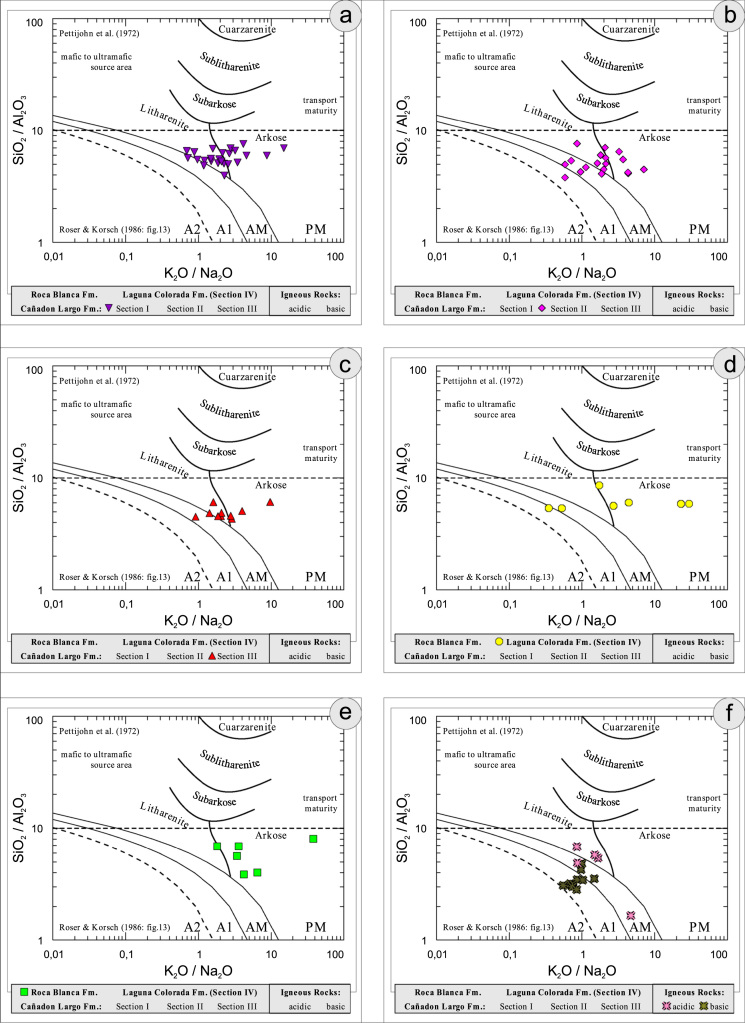
Geochemical sediment classification of El Tranquilo sediments and igneous rocks: K_2_O/Na_2_O–SiO_2_/Al_2_O_3_ after [1].

**Fig. 10:**
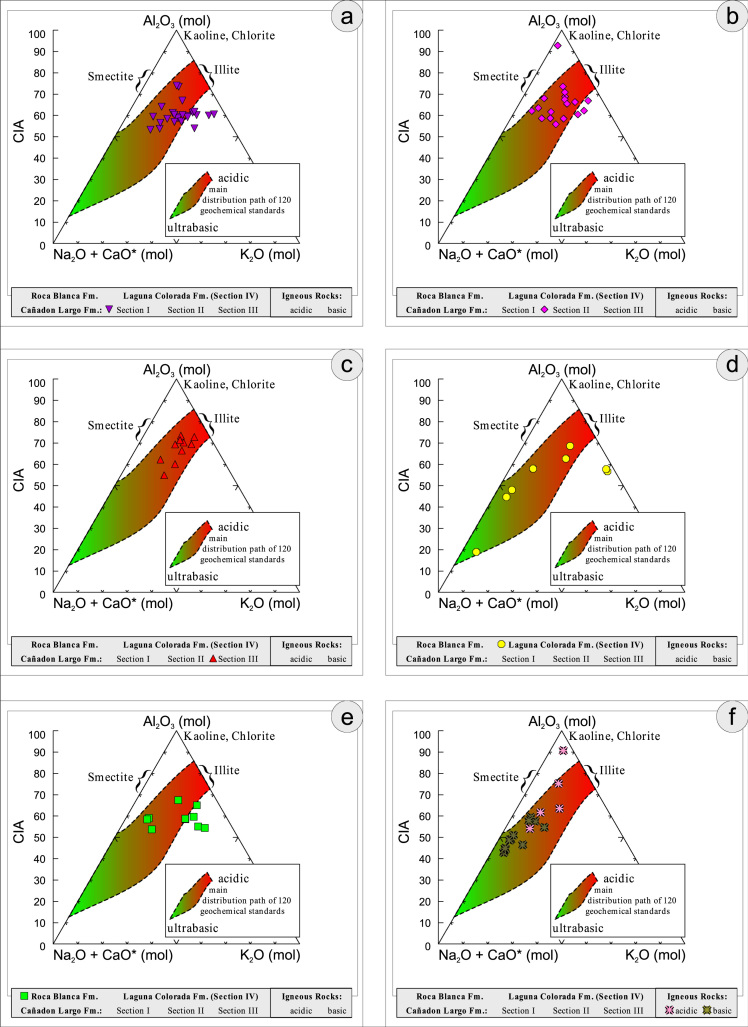
Geochemical sediment classification of El Tranquilo sediments and igneous rocks: Na_2_O+CaO*/Al_2_O_3_/K_2_O after [11] modified by [1].

**Fig. 11:**
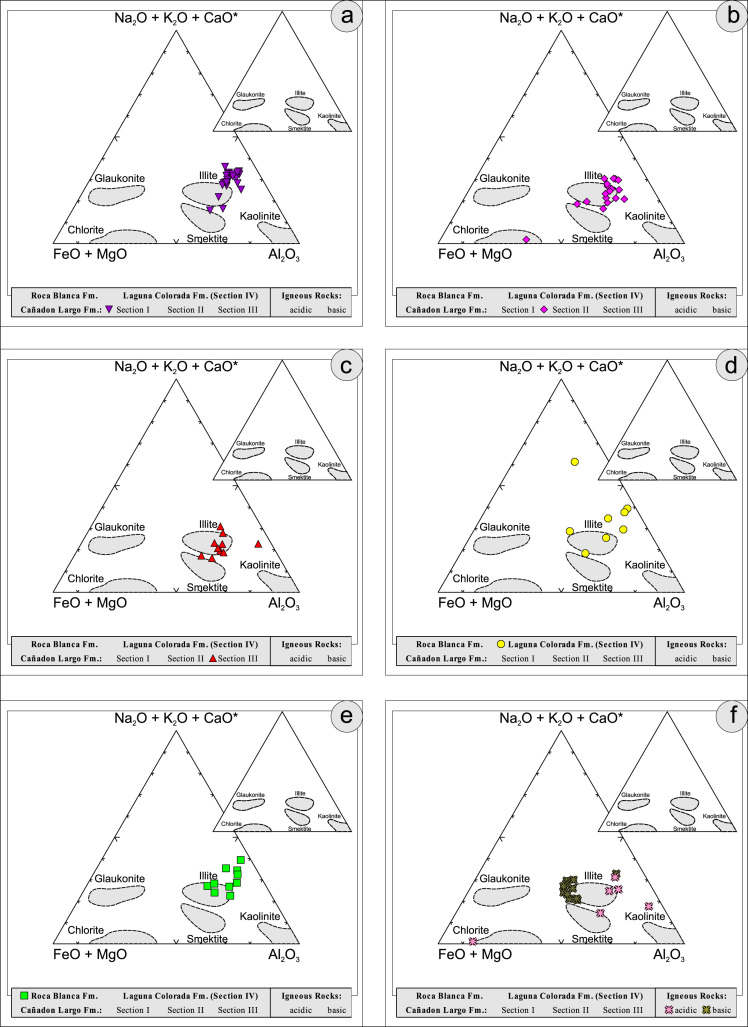
Geochemical sediment classification of El Tranquilo sediments and igneous rocks: FeO+MgO/Na_2_O+K_2_O+CaO*/Al_2_O_3_ after [12].

**Fig. 12:**
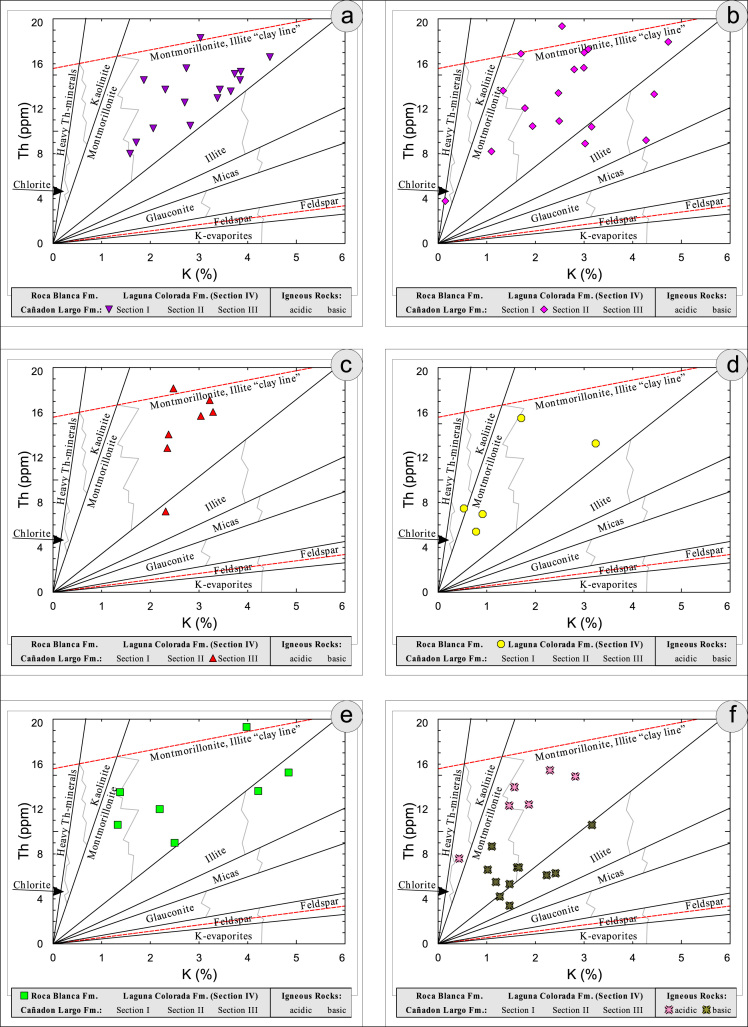
Geochemical sediment classification of El Tranquilo sediments and igneous rocks: K/Th after [13].

**Fig. 13:**
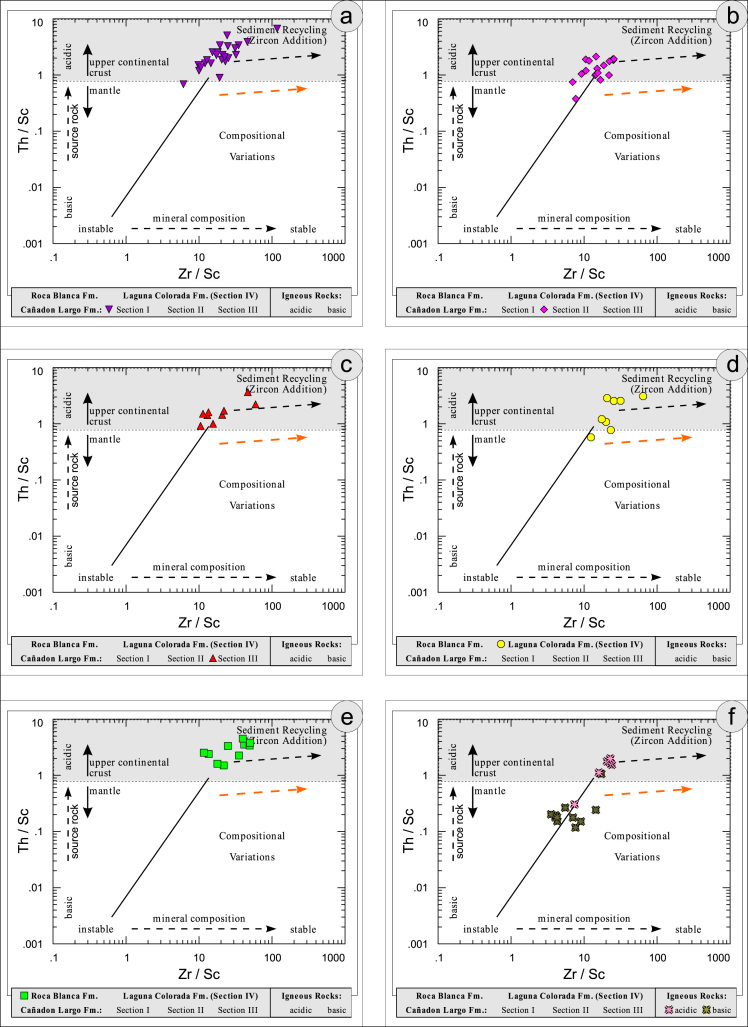
Geochemical sediment classification of El Tranquilo sediments and igneous rocks: Zr/Sc–Th/Sc diagram after [14] modified by [1].

**Fig. 14:**
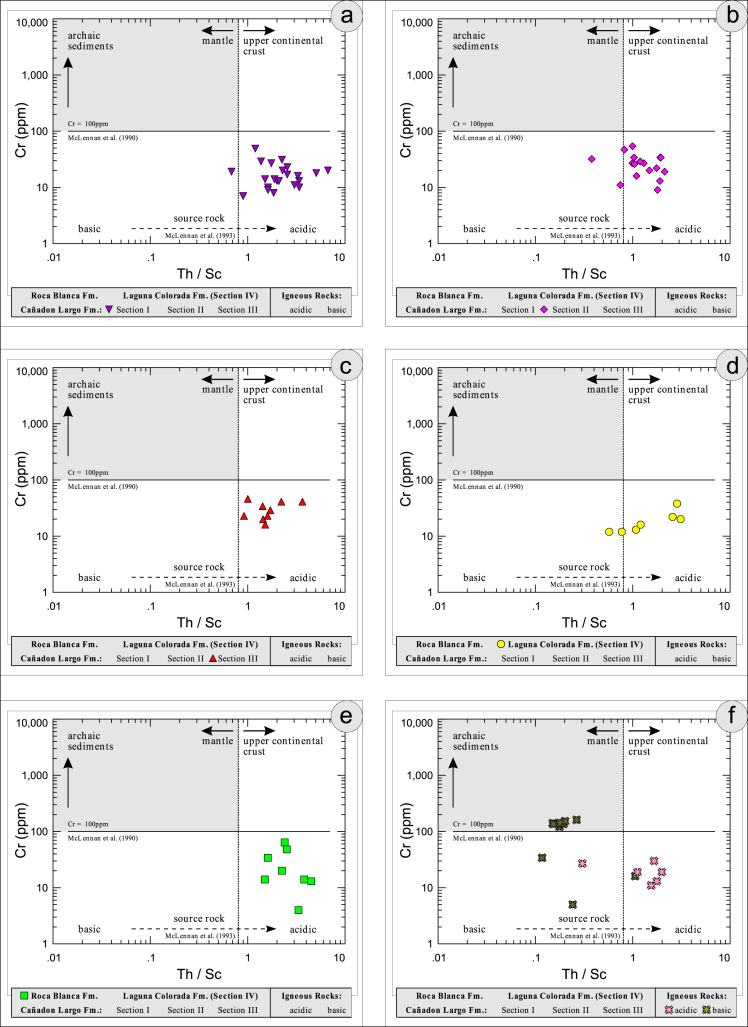
Geochemical sediment classification of El Tranquilo sediments and igneous rocks: Th/Sc–Cr after [1].

**Fig. 15:**
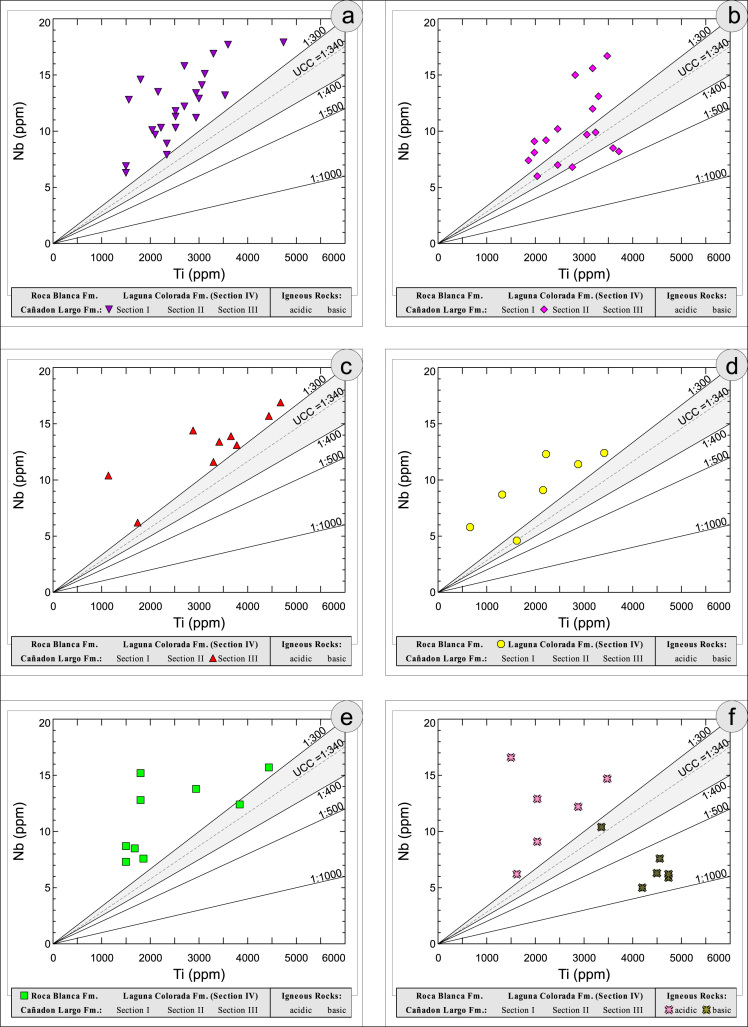
Geochemical sediment classification of El Tranquilo sediments and igneous rocks: Ti–Nb after [15].

**Fig. 16:**
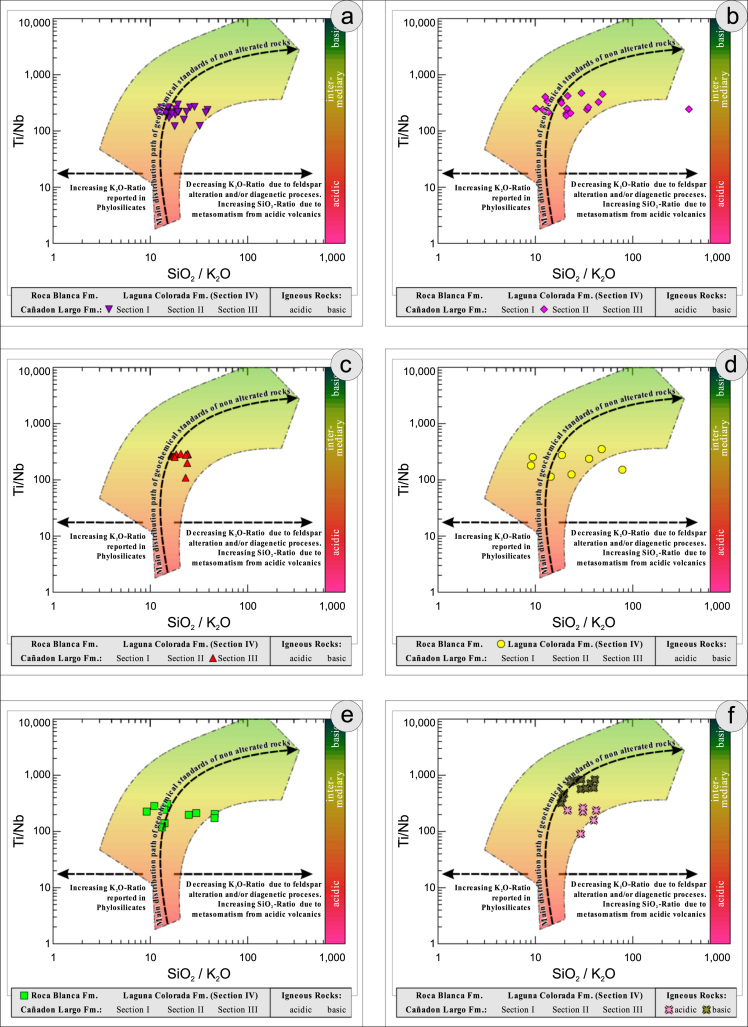
Geochemical sediment classification of El Tranquilo sediments and igneous rocks: SiO_2_/K_2_O–Ti/Nb after [1].

**Fig. 17:**
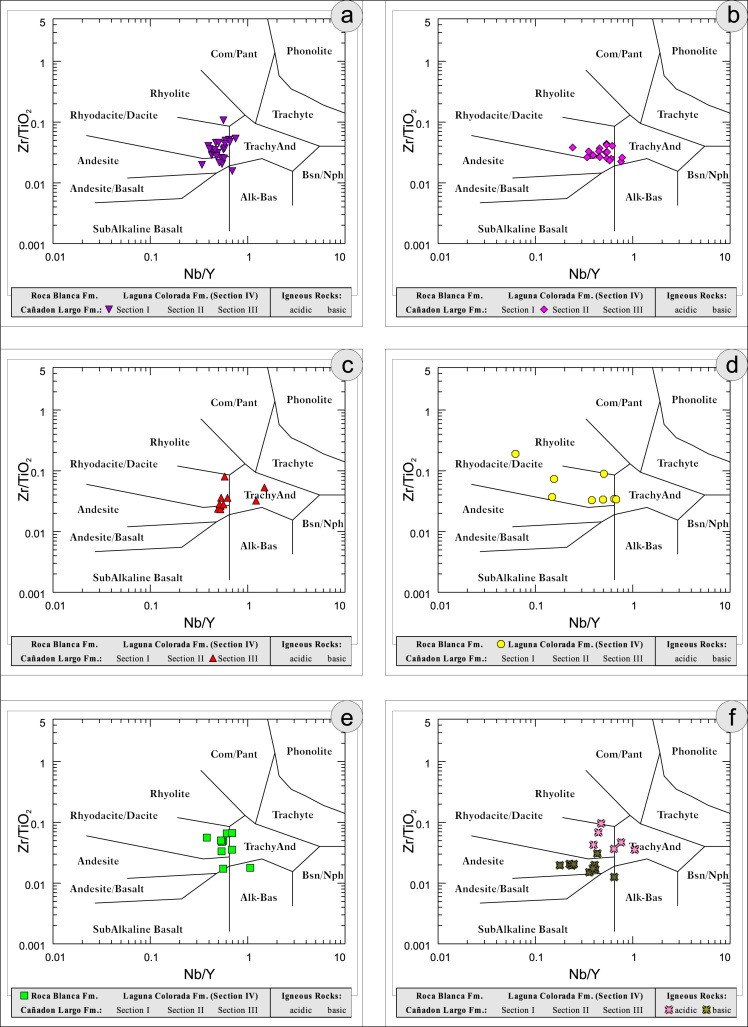
Geochemical sediment classification of El Tranquilo sediments and igneous rocks: Nb/Y–Zr/TiO_2_ after [16].

All the geochemical data were plotted separately for the different sections into the following diagrams SiO_2_/Al_2_O_3_ after [9] (Fig. 7); K_2_O/Na_2_O [10], modified by [1] (Fig. 8); K_2_O/Na_2_O–SiO_2_/Al2O_3_ [1] (Fig. 9); Na_2_O + CaO* vs. Al_2_O_3_ vs. K_2_O, after [11], [19] modified by [1] (Fig. 10); Na_2_O+K_2_O+CaO vs. FeO+MgO vs. Al_2_O_3_ [12] (Fig. 11); K/Th [13] (Fig. 12); Th/Sc–Zr/Sc ratio [14] (Fig. 13); Th/Sc–Cr [1] (Fig. 14); Ti/Nb [15] (Fig. 15); Ti/Nb–SiO_2_/K_2_O [1] (Fig. 16); Nb/Y–Zr/TiO_2_ [16] (Fig. 17).
