# Supplementary material for: Petrological and geochemical (major-, trace-, and rare earth element) data of the Triassic El Tranquilo Group, Deseado Massif, Patagonia, Argentina
Source: Data Brief. 2018 Nov 16;21:1970–2014. doi: 10.1016/j.dib.2018.11.062 (PMC6258889; doi:10.1016/j.dib.2018.11.062)
Supplement: Supplementary file 1 — Supplementary material. [file mmc1.pdf]

UANL
UNIVERSIDAD AUTÓNOMA DE NUEVO LEÓN®

FCT
FACULTAD DE CIENCIAS DE LA TIERRA®

Ex-Hacienda de Guadalupe, 08 de noviembre de 2018

AUTHOR DECLARATION TEMPLATE

I wish to confirm that there are no known conflicts of interest associated with this publication and there has been no significant financial support for this work that could have influenced its outcome.

I confirm that the manuscript has been read and approved by all named authors and that there are no other persons who satisfied the criteria for authorship but are not listed. I further confirm that the order of authors listed in the manuscript has been approved by all of us.

I confirm that we have given due consideration to the protection of intellectual property associated with this work and that there are no impediments to publication, including the timing of publication, with respect to intellectual property. In so doing I confirm that we have followed the regulations of my institution concerning intellectual property.

I understand that the Corresponding Author is the sole contact for the Editorial process (including Editorial Manager and direct communications with the office). I am responsible for communicating with the other authors about progress, submissions of revisions and final approval of proofs.

Signed by all authors as follows:

Dr. Uwe Jenchen

Profesor Titular "B", T.C.

Uwe.jenchen@gmail.com

c.c.p.:archivo

Dr. Uwe Jenchen
Diplom-Geologe
Carr. a Cerro Prieto Km. 8 Ex-hacienda de Guadalupe, C.P. 67700, A.P. 104
Linares, Nuevo León, México.
Teléfonos y Fax (821) 2142010, 2142020, 2142030, ext 3851
www.fct.uanl.mx e-mail: uwe.Jenchen@gmail.com
